# Exploiting orthology and de novo transcriptome assembly to refine target sequence information

**DOI:** 10.1186/s12920-019-0524-5

**Published:** 2019-05-23

**Authors:** Julia F. Söllner, Germán Leparc, Matthias Zwick, Tanja Schönberger, Tobias Hildebrandt, Kay Nieselt, Eric Simon

**Affiliations:** 1 Computational Biology & Genomics, Boehringer Ingelheim Pharma GmbH & Co. KG, Birkendorfer Strasse 65, 88397 Biberach an der Riss, Germany; 2 Transl. Medicine + Clin. Pharmacology, Boehringer Ingelheim Pharma GmbH & Co. KG, Birkendorfer Strasse 65, 88397 Biberach an der Riss, Germany; 3 Drug Discovery Sciences, Boehringer Ingelheim Pharma GmbH & Co. KG, Birkendorfer Strasse 65, 88397 Biberach an der Riss, Germany; 4 Integrative Transcriptomics, Center for Bioinformatics, University of Tübingen, Sand 14, 72076 Tübingen, Germany

**Keywords:** RNA-Seq, de novo transcriptome assembly, Orthology, Sequence refinement, Comparative genomics

## Abstract

**Background:**

The ability to generate recombinant drug target proteins is important for drug discovery research as it facilitates the investigation of drug-target-interactions in vitro. To accomplish this, the target’s exact protein sequence is required. Public databases, such as Ensembl, UniProt and RefSeq, are extensive protein and nucleotide sequence repositories. However, many sequences for non-human organisms are predicted by computational pipelines and may thus be incomplete or incorrect. This could lead to misinterpreted experimental outcomes due to gaps or errors in orthologous drug target sequences. Transcriptome analysis by RNA-Seq has been established as a standard method for gene expression analysis. Apart from this common application, paired-end RNA-Seq data can also be used to obtain full coverage cDNA sequences via de novo transcriptome assembly.

**Methods:**

To assess whether de novo transcriptome assemblies can be used to determine a protein’s sequence by searching the assembly for a known orthologous sequence, we generated 3 × 6 = 18 tissue specific assemblies (three organs: brain, kidney and liver; six species: human, mouse, rat, dog, pig and cynomolgus monkey). These assemblies and the manually curated human protein sequences from UniProtKB/Swiss-Prot were used in a reciprocal BLAST search to identify best matching hits.

We automated and generalised our approach and present the a&o-tool, a workflow which exploits de novo **a**ssemblies of paired-end RNA-Seq data and **o**rthology information for target sequence validation and refinement across related species. Furthermore, the a&o-tool extracts best hits’ sequences from a reciprocal BLAST search, translates them into protein sequences, computes a multiple sequence alignment and quantifies the refinement.

**Results:**

For the three human assemblies we observed a hit rate greater than 60% with 100% sequence coverage and identity. For assemblies from the other species we observed similar hit rates and coverage with highest identities for cynomolgus monkey.

**Conclusions:**

In summary, we show how to refine protein sequences using RNA-Seq data and sequence information from closely related species. With the a&o-tool we provide a fully automated pipeline to perform refinement including cDNA translation and multiple sequence alignment for visual inspection. The major prerequisite for applying the a&o-tool is high quality sequencing data.

**Electronic supplementary material:**

The online version of this article (10.1186/s12920-019-0524-5) contains supplementary material, which is available to authorized users.

## Background

During drug development a compound’s efficacy and safety have to be shown in a non-human species before it can proceed to clinical trials where it is eventually tested in humans. Particularly the selection of the right pharmacological dose which is required for target engagement is crucial for the interpretation of experimental results. The appropriate dose, in turn, is closely linked to the compound’s activity. Fortunately, biochemical and cellular in vitro assays can be used throughout the drug discovery process to assess the compound’s activity on the target protein. This can be accomplished by using cell lines or bacteria expressing the recombinant protein, i.e. a DNA template of the known target sequence which is to be introduced into the production system. However, erroneous target proteins lead to an over- or underestimation of the compound’s activity or wrong dose selection and subsequently to misinterpretation of in vivo experiments.

**Fig. 1:**
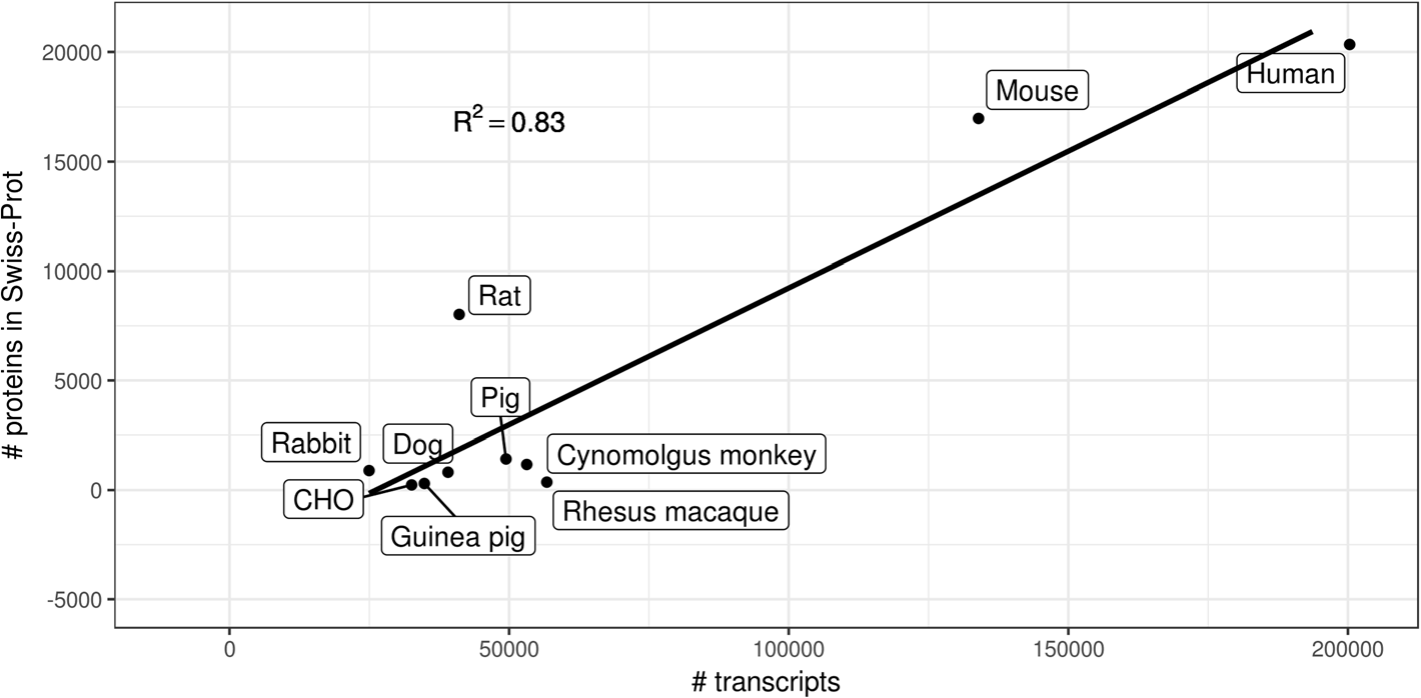
Comparison of the number of transcripts annotated in Ensembl and the number of protein sequences in Swiss-Prot (accessed on: 26/04/2018) in species that are relevant for pharmaceutical research. CHO: Chinese Hamster Ovary cells

Public databases, such as Ensembl [1], UniProt [2] and RefSeq [3], provide genome-wide sequence information for most model organisms commonly used in pharmaceutical research. However, not all genomes of these species are annotated equally well. When comparing the number of transcripts reported in Ensembl [1] (version 91) to the number of proteins with manually reviewed sequences in UniProtKB/Swiss-Prot [2] (accessed: 26/04/2018) one can see that the human genome has the highest number of annotated transcripts as well as the highest number of curated protein sequences (see Fig. 1). Since the house mouse is an established model organism it is investigated almost as thoroughly as humans, i.e., there are almost as many manually curated sequences as for human. In contrast, protein sequences from some relevant, closely related non-human primate species, like cynomolgus monkey (*Macaca fascicularis*) and rhesus macaque (*Macaca mulatta*), are not very well curated. This is also indicated by the fact that the Ensembl reference genome for cynomolgus monkey has been released only recently (Ensembl version 91 in December 2017). Another example is the Chinese hamster genome which is still poorly annotated although Chinese hamster ovary cells (CHO) are important vector systems for the production of biopharmaceuticals, like therapeutic monoclonal antibodies.

Even though the sequence information provided in the databases named above are generated with a combination of elaborate automated pipelines and manual curation, the sequences may still be incomplete or contain errors. The NCBI automated genome annotation pipeline, for example, aligns known transcripts, proteins (including ones from other species if available) and existing RNA-Seq reads to the corresponding reference genome and integrates this information into their gene prediction models [4]. Well-studied species like human will therefore have a more reliable genome annotation due to the larger amount of available data.

We want to build upon the principle of the automated annotation pipelines and exploit orthology relationships and RNA-Seq data to refine the sequence of a target protein with presumably poor or incomplete sequence information. The a&o-tool performs de novo transcriptome assembly from RNA-Seq data of the species of interest and uses a known bait sequence from a closely related species to identify the best matching contig for a given target protein. The best matching contig is then searched for open reading frames and translated into an amino acid sequence. To assess the quality of the resulting protein sequence we perform a multiple sequence alignment and compute statistics based on pairwise alignments.

There are already tools making use of transcriptome assemblies, however, they are not focusing on the sequence of a specific target but on the prediction of the exon-intron-structure. One example is Scipio [5] which aligns a query protein to a genome sequence to determine the gene’s structure including intron-exon boundaries and splice sites. Another tool, MIKADO [6], integrates several transcriptome assemblies to improve transcript models. While the main emphasis of these tools is on the overall gene structure annotation, the a&o-tool is designed and optimised to provide target-centric coding sequence information by making use of well-annotated orthologous sequences.

The paper is structured as follows: We first present an estimate on how many sequences might benefit from our refinement approach in five pharmaceutical model species. Next, we validate the general idea of refining poorly annotated protein sequences by aligning the known protein sequences from human to de novo assemblies from three tissue-specific transcriptomes (brain, liver and kidney) of these species. For this purpose, we use the 20,350 manually reviewed human protein sequences in UniProtKB/Swiss-Prot (hereafter referred to as “known human protein sequences”) as reference sequences. The Swiss-Prot subset of the UniProtKB/Swiss-Prot database [2] is probably the most comprehensive resource for curated protein sequences. The number of human entries in this database has been quite stable for almost a decade indicating that most human proteins are known. We generalise the approach used during validation of the general idea with an automated sequence refinement workflow implemented in the a&o-tool and show an example application. For our analyses we used both publicly available data (mouse, rat, dog, pig and human) as well as newly generated paired-end RNA-Seq data (cynomolgus monkey).

## Methods

### Data description

We used paired-end RNA-Seq raw read data from two publicly available and one in-house data sets (see Table 1). Fushan et al. [7] have published data for mouse, rat, dog and pig amongst several other mammalian species. For their study they sequenced samples from brain, kidney and liver. Human RNA-Seq data were retrieved from the Human Protein Atlas [8, 9]. The in-house data from cynomolgus monkey are described below. A list of all samples can be found in Additional file 1. Details on sample preparation and sequencing of the two publicly available data sets are described in the original publications. Curated protein sequences from UniProtKB/Swiss-Prot for human, mouse, rat, dog, pig and cynomolgus monkey were downloaded on June 9th, 2018.

**Table 1:** RNA-Seq data sets used for the analyses

Species	Data source	RNA-Seq Details
Human	Uhlén et al. [8, 9]	17 ×10^6^ reads per sample
Mouse, rat, dog, pig	Fushan et al. [7]	15 ×10^6^ reads per sample
Cynomolgus monkey	this study	RIN median 8.7, 2x85bp on HiSeq3000, ca. 55 ×10^6^ reads per sample

### RNA-Seq of cynomolgus monkeys

RNA has been isolated from brain, kidney and liver tissue samples of two approximately ten years old female cynomolgus monkeys (*Macaca fascicularis*). Treatment of the animals followed the German Law on the Protection of Animals and was performed with permission of the regional authorities. Per animal and tissue three samples were collected. The cynomolgus monkeys were held in an AAALAC-accredited facility, group-housed (males and females) in stainless steel cages with free access to an outside “open-air” enclosure and were fed a standard primate diet with additional fresh fruit and had access to water ad libitum. For environmental enrichment the animals had access to toys, wooden bricks, puzzle feeder and a swimming pool. The animals were anesthetized with ketamine (10 mg/kg b.w.) and midazolam (0.1 mg/kg b.w.) and subsequently euthanized by intravenous injection of pentobarbital (100 mg/kg b.w.). Tissues were harvested and transferred immediately to RNA Later at 4 °C.

For each tissue sample, total RNA was extracted using the Ambion Magmax™-96 total RNA isolation kit (Life Sciences). Accordingly, 5 mg of tissue was preserved in lysis solution and subsequently homogenized 15 s in PeqLabs (now VWR) Precyllys, twice. Nucleic acids were then captured using magnetic beads, washed and incubated with DNase. Finally, total RNA was eluted in 50 μl elution buffer and checked and quantified with the Fragment Analyzer from AATI (now Agilent) using the total RNA Standard Sensitivity protocol.

The sequencing library preparation has been done using 200 ng of total RNA input with the TruSeq® Stranded mRNA LT - Set B (RS-122-2102, Illumina Inc., San Diego, CA) producing on average 275 bp fragments including adapters. We generated seven libraries and pooled them after normalization using the adapter indices supplied by the manufacturer. We clustered the pooled libraries on the cBot Instrument from Illumina using the HiSeq® 3000/4000 PE Cluster Kit - cBot - PE-410-1001 Illumina Inc., San Diego, CA. Finally, paired-end sequencing was performed as 2x85bp with a seven bases index read on an Illumina HiSeq3000 instrument pooling two Kits of FC-410-1001, HiSeq® 3000/4000 SBS Kit (50 cycles) Illumina Inc., San Diego, CA.

### RNA-Seq data mapping, counting and normalisation

FastQC v0.11.2 [10] was used to investigate the quality of sequenced reads. We used STAR v2.5.2a11 [11] to align the RNA-Seq reads to the respective species’ reference genome (Ensembl version 92). Duplication rates in the samples were determined with bamUtil v1.0.11 [12] and assessed with the dupRadar v1.4 Bioconductor R package [13]. We used Cufflinks software version 2.2.114 to quantify gene expression as Fragments Per Kilobase of transcript per Million mapped reads (FPKM). Furthermore, we used featureCounts [14] to obtain read counts. We used MultiQC [15] v1.3 to summarise the quality metrics generated during the analyses. A detailed description of the analysis pipeline is given in [16]. All data sets listed in Table 1 were processed in single batches from independent experiments. We investigated potential confounding factors by principal component analysis (PCA). However, we did not observe any confounding effects in the major components of the PCA. In all experiments, there is a strong clustering by tissue origin (see Additional file 2: Figures S1–S6) in the first principal component.

### Proportion of genes to be improved

To estimate the number of incomplete sequences we used the biomaRt package [17, 18] (version 2.32.1) with Ensembl version 92 to query information on human genes having an orthologous gene in either of the five other species (mouse, rat, dog, pig and cynomolgus monkey). In particular, we fetched the sequence identities based on amino acid alignments, i.e. the percentage of orthologous sequence matching the human sequence (target identity) and the percentage of human sequence matching the orthologous sequence (query identity). By subtracting the query identity from the target identity, we obtained a difference in sequence identity which we used to detect non-human genes that are shorter or longer than their human orthologue. The rationale behind this approach is that if, for example, 98% of the orthologous sequence matches the human sequence in a protein alignment (referred to as target identity in Ensembl) but only 80% of the human sequence matches the orthologous sequence (referred to as query identity in Ensembl), the orthologous protein sequence might be incomplete because of a missing fragment. Vice versa, if the query identity is significantly higher than the target identity the orthologous protein sequence might be too long because e.g. the correct stop or splice site is missing.

We computed the mean and standard deviation of these differences for each species and marked genes as potentially not perfectly annotated if their absolute difference in sequence identity was greater than the species’ mean + 2 times standard deviation. We chose this conservative threshold based on the distribution of the differences in sequence identities (see Additional file 2: Figures S7–S11) to obtain the set of proteins with the most significant deviation. To ensure that the high difference in sequence identity reflects poor annotation instead of an evolutionary divergence, we have further analysed only those genes when their sequence appears to be well conserved across the other four species. This was done by filtering for genes that have a high difference in sequence identity in only one species but not the others.

### De novo transcriptome assemblies

We computed tissue-specific de novo transcriptome assemblies with BinPacker [19] (version v1.0) and rnaSPAdes which is part of the SPAdes package [20] (version 3.11.1) using default parameters.

A comparison of the two assemblers based on TransRate [21] metrics shows that SPAdes suffers from a large proportion of short (< 200 bp) contigs. Furthermore, BinPacker lead to a slightly higher coverage of the known human sequence in our validation (see Additional file 2: Figures S13–S18). Due to these observations we decided to use BinPacker for our analyses and in the automated pipeline.

### Evaluation of detection rates in human and related species from RNA-Seq assemblies

To assess whether RNA sequencing data can reliably improve sequence information, we used assemblies from human RNA-Seq data and compared them to the canonical isoforms of all known human proteins according to UniProtKB/Swiss-Prot. We think that this is a reasonable approach since the canonical sequence for a given human protein as reported by UniProtKB/Swiss-Prot corresponds to the most frequent or most conserved protein isoform in orthologous species. To identify the best contig for a given human protein target we performed a reciprocal best BLAST search: First, we aligned each known human sequence to all assembly contigs (using tblastn, NCBI BLAST+ [22] version 2.7.1), to determine the best matching contig. Then we aligned the best hits back to all known human sequences (using blastx, NCBI BLAST+ version 2.7.1). Based on these results we calculated the rate of known human proteins for which we received the correct human protein as best hit in the backward blastx search.

Gene expression levels of proteins which were found as best reciprocal hit (RBH) and those that were not (non-RBH), were compared to investigate whether the non-RBH proteins were lowly expressed. Human UniProt accession numbers were mapped to human Ensembl gene identifiers via the biomaRt R package using Ensembl (version 92). biomaRt was also used to retrieve orthologous Ensembl gene identifiers.

Furthermore, we investigated the sequence identity reported by BLAST and the percentage of the human protein sequence covered by the alignment. The coverage was computed from the alignment start and end positions in the human protein and the protein’s length.

The reciprocal best BLAST procedure described above was also applied to mouse, rat, dog, pig and cynomolgus monkey. The database for the initial tblastn search was constructed from the assembly of the respective species and known human protein sequences were aligned to it. The best hits were then aligned back to the database of all known human protein sequences using blastx.

### Generalised refinement pipeline

The reciprocal best hit BLAST search described in the previous section is restricted to use cases where a well curated reference set of protein sequences is available and the target sequence in the assembly is complete and highly similar to the bait sequence.

**Fig. 2:**
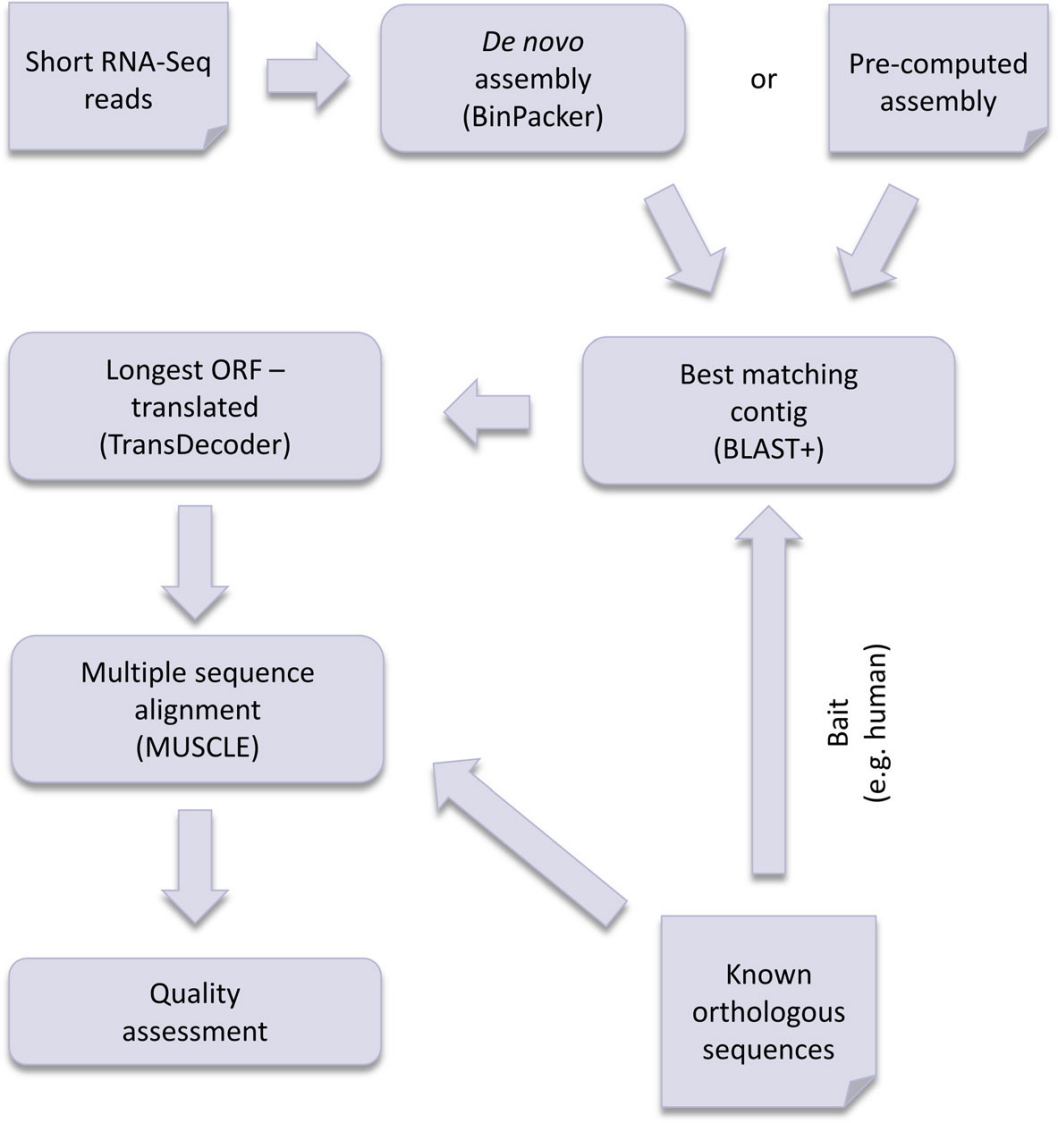
Schematic overview of the Nextflow sequence refinement pipeline, the a&o-tool. All required input, i.e. RNA-Seq data or pre-computed assembly, orthologous protein sequences in FASTA format and the name of the bait species are passed via a JSON file

For processing an arbitrary target-bait sequence pair (e.g. using a non-human bait sequence), we provide a generalised and automated workflow, the a&o-tool (see Fig. 2).

This workflow relies on two inputs: Good quality, paired-end raw read data (or a high-quality transcriptome assembly) from an RNA-Seq experiment in which the target protein transcript is expressed at reasonable levels and the orthologous sequence (bait).

We begin with constructing a de novo transcriptome assembly from paired-end RNA-Seq reads applying BinPacker (version v1.0) with the programme’s default parameters. Since the assembly process is computationally intense, we recommend pre-computing the assembly for repeated querying of the same RNA-Seq data set. The assembly can be passed to the pipeline via the configuration file.

The orthologous protein sequence is then aligned to the transcriptome assembly using tblastn (NCBI BLAST+ [22] version 2.7.1). To determine which assembled contiguous sequences (contigs) match the orthologous sequence best, we sort the BLAST result by bitscore and e-value. Based on this we choose the *n* best matching contigs and fetch their cDNA sequences from the assembled transcriptome.

Applying TransDecoder [23], these cDNA sequences are searched for open reading frames (ORFs) and translated into amino acid sequences. For each of the *n* contigs we select the longest ORF as its protein representation. The resulting protein sequence is validated in two different ways. A multiple sequence alignment (MSA) of the predicted sequence(s) and orthologous proteins allows for visual assessment of refinement success. The MSA is generated using MUSCLE [24]. The annotated target sequence can also be provided with the input set of orthologous sequences for a comparison with the refined bait and all other homologous sequences.

The refinement is quantified by the difference of pairwise sequence identities of the existing target vs. bait sequence and refined target vs. bait sequence. In case there is no annotated sequence we compute the difference in percentage of predicted sequence covered by the alignment to the orthologous bait and the percentage of the bait sequence covered.

To automate the workflow described above it was implemented in Nextflow [25]. The input for the pipeline script is a configuration file in JSON format. The user can either pass the path to a pre-computed assembly or the path to raw read data from paired-end experiments. Furthermore, a file with the orthologous bait sequence and other sequences to be included in the multiple sequence alignment have to be provided in FASTA format. Apart from BinPacker, BLAST+ and MUSCLE, the Nextflow script contains bash and Python code. The required software is provided via a Docker [26] container and the pipeline as well as example data are available on GitHub (https://github.com/Julia-F-S/a-o-tool).

## Results

### Proportion of genes to be improved

Across the five investigated species the number of affected genes ranged from 474 in dog (3% of all dog genes reported as one-to-one orthologues to human) to 259 in mouse, i.e. 1.5% of all mouse genes reported as one-to-one orthologues to human (see Additional file 2: Figure S12).

### De novo transcriptome assemblies

We assembled 18 tissue-specific transcriptomes (six species times three tissues brain, liver and kidney) with the de novo assembler BinPacker. A quantitative summary of the assembled transcriptomes was generated by TransRate (see Table 2 for human and Additional file 3 for the other species). For the human tissue-specific assemblies the mean contig length is 1369 bases (mean across assemblies from the three tissues). Furthermore, 29.2% of the contigs (again mean across all three tissues) contain an open reading frame (ORF). In contigs with an ORF, the mean percentage of the contig being covered by the ORF is 43.1%.

**Table 2:** Quantitative metrics for the human tissue-specific assemblies computed by TransRate

	brain	liver	kidney
# contigs	165,810	88,083	128,530
max. Contig length [bp]	30,531	27,458	23,822
mean contig length [bp]	1367.92	1383.32	1355.58
# contigs with ORF	42,581	29,226	37,009
coverage of contigs with ORF [%]	39.8	45.9	43.6

The number of contigs, the number of base pairs in the longest contig, the mean contig length and the number of ambiguous bases (# N bases) provide information on the basic characteristics of the assembly. The number of contigs containing an open reading frame (# contigs with ORF) and the mean percentage of the contig being covered by the ORF (coverage of contigs with ORF [%]) help to assess the protein-coding potential of the assembled contigs

### Evaluation of detection rates of human and orthologous coding sequences from RNA-Seq assemblies

**Fig. 3:**
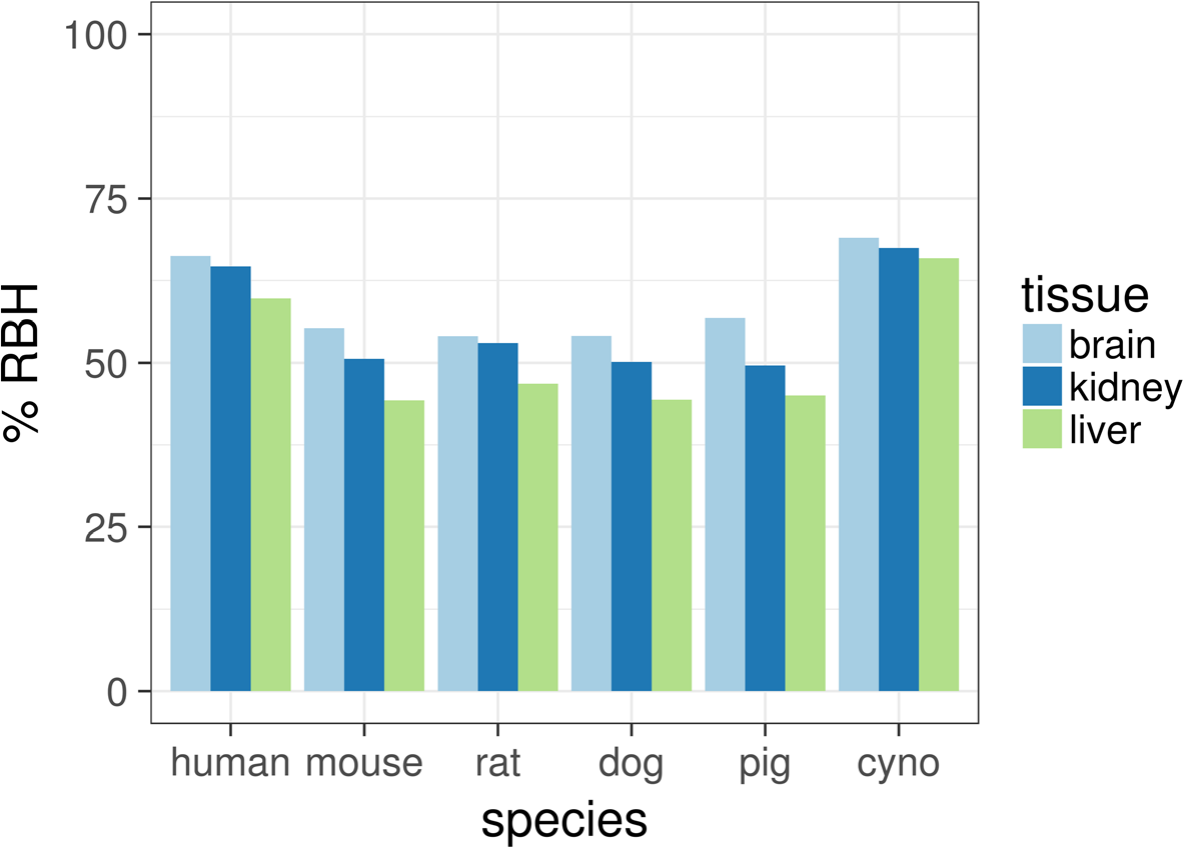
Proportion of human proteins with a reciprocal best hit in the respective assembly. The percentage on the y-axis refers to all 20,350 human proteins. Each bar represents the result from the search with the assembled transcriptome in the respective species and tissue

To assess whether RNA sequencing data can be reliably used to improve sequence information, we followed a reciprocal best hit (RBH) BLAST approach. When searching for all known human protein sequences in the tissue specific assemblies of human RNA-Seq data, we were able to obtain a reciprocal best BLAST hit for 63.6% of all known proteins (mean across tissues; see Fig. 3). One should note that the initial tblastn search revealed significant alignments (e-value <1e^− 4^) for 18,906 of all 20,350 human proteins (93%). However, a large proportion of the corresponding contigs did not report the query protein sequence as the significant top hit of the reverse blastx search. Interestingly, the observed per tissue detection rate of 64% is relatively close to the proportion of protein coding genes that we could robustly detect by the RNA-Seq method: For example, in the human expression data we observed on average 14,265 genes with an FPKM greater or equal to 1, a lower threshold for genes considered as expressed [9]. In relation to the 20,350 human proteins in UniProtKB/Swiss-Prot this corresponds to 70% expressed protein coding genes.

Performing a reciprocal BLAST search of the assembled transcriptomes from mouse, rat, dog and pig with the known human protein sequences lead to about 50% RBHs, i.e. fewer than a reciprocal search with the human transcriptome assembly and human proteins. Compared to the combination of human assembly and human transcriptome, using the cynomolgus monkey assemblies and human proteins resulted in a slightly higher hit rate (mean increase of 3.9% across tissues).

Comparing the results between tissues, we observed a consistent pattern across all species: The percentage of RBHs in brain assemblies was highest, in kidney assemblies it dropped by 1.6% and in liver assemblies there was a decrease of 6.5% in comparison to assemblies from brain data (see Fig. 3).

**Fig. 4:**
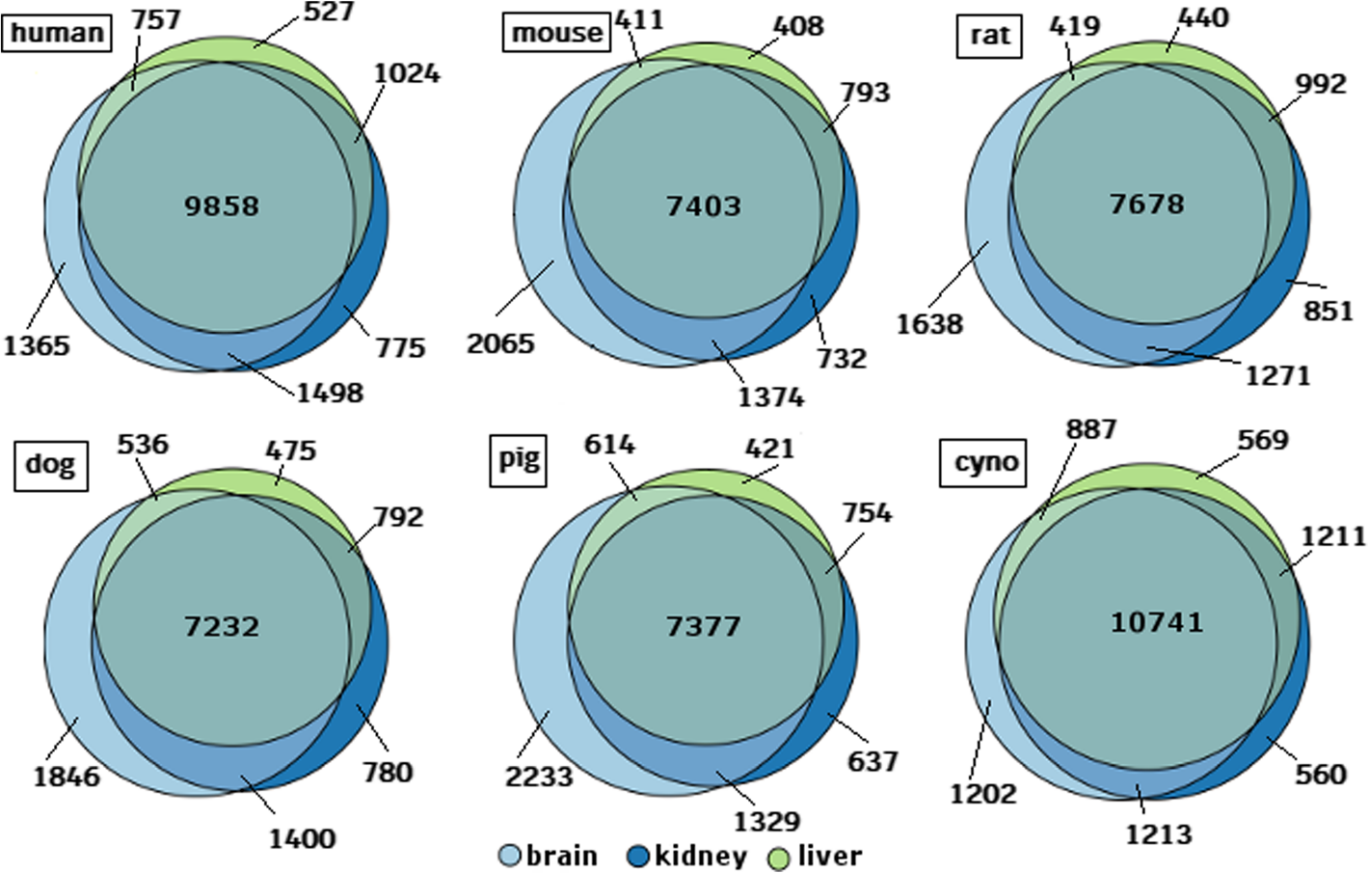
Area-proportional Venn diagram [30] of the tissue-specific sets of human proteins having an RBH in human, mouse, rat, dog, pig and cynomolgus monkey (cyno) assemblies. Colours represent different tissues

We also looked at the intersection of RBH proteins across the three tissues for each species to determine whether there are tissue-specific RBHs (see Fig. 4). While for all six species the majority of proteins is detected in all three tissues (from 7232 in pig to 10,741 in cynomolgus monkey), there is also a significant number of tissue-specific RBHs. Across all species the most tissue-specific hits are found in assemblies from brain RNA-Seq reads (> 1200), followed by those from kidney (> 500) and liver (> 400). In human, the union set of all RBHs covers 77.7% of all known human proteins, the RBHs in mouse, rat, dog and pig correspond to 65% of the known human proteins (mean across species) and the union of all RBHs in cynomolgus covers 80.5% of all human proteins.

To assess the quality of the reciprocal best hits in more detail, we computed the percentage of the human sequence which was covered by the alignment with the best hit contig as well as the sequence identity. In general, HSPs yielded good coverage across all species (see empirical cumulative coverage distributions in Additional file 2: Figures S13–S18). In human more than 90% of all HSPs showed coverage greater than 50% of the known human protein and almost 60% of all HSPs covered more than 95% of the respective known human protein. For the alignments of non-human assemblies and human sequences these numbers do not deviate significantly.

The cumulative density of sequence identity of the HSPs indicates that overall the resulting HSPs are highly identical to the part of the human protein which is covered by the alignment (see Additional file 2: Figures S19–S24). In human, more than 96% of HSPs have a sequence identity greater than 90%. In the two rodent species about 50% of all HSPs show more than 90% sequence identity while in dog and pig roughly 60% are more than 90% identical to the corresponding human protein. The cynomolgus monkey sequences are more similar to human, as more than 90% of the HSPs assembled sequences are more than 90% identical to the human sequence.

**Fig. 5:**
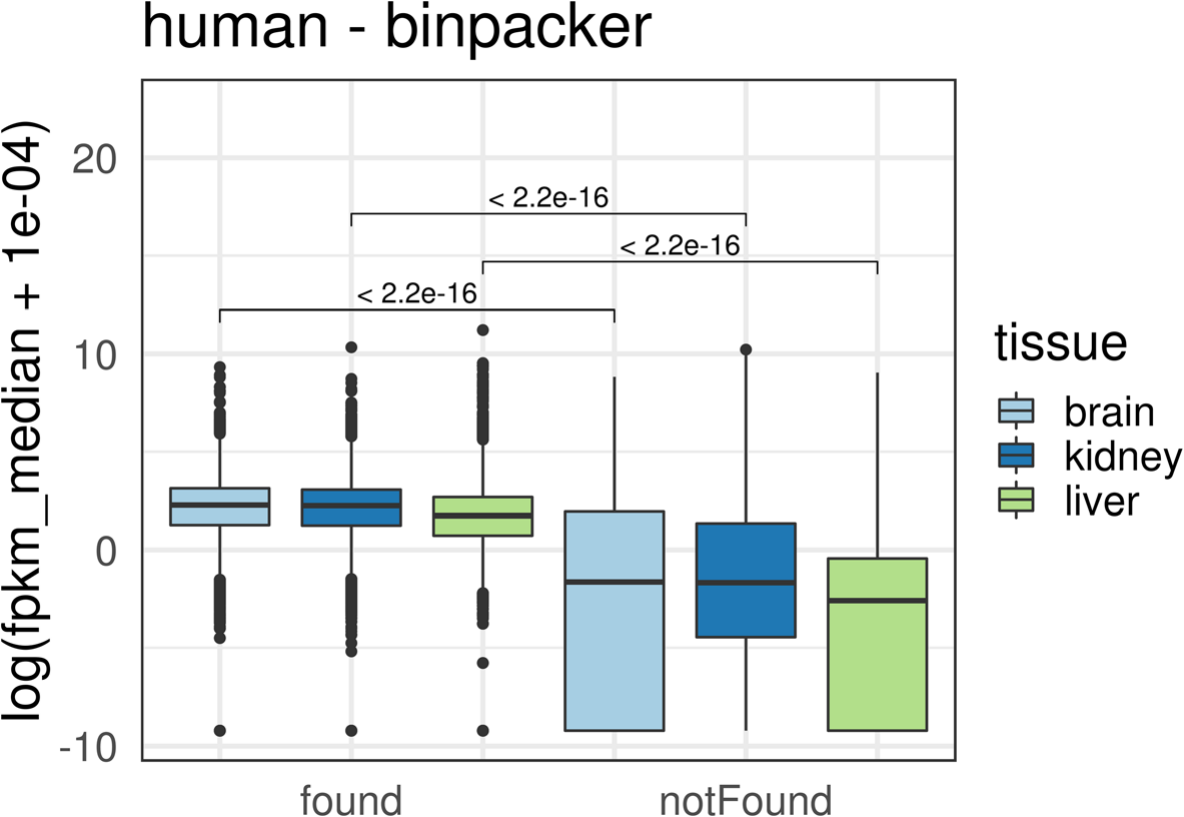
Expression levels between proteins with a reciprocal best BLAST hit (found) and those without (notFound). Tissue-specific pairwise significance has been determined with a Wilcoxon rank sum test

We hypothesised that proteins which are not found as an RBH are lowly or not at all expressed and thus are not present in the assembled transcriptome. This was confirmed by comparing the distribution of expression levels in the group of proteins found as reciprocal best hits and proteins not being an RBH (Wilcoxon rank sum test: *p*-value <2e-16 for all tissues; see Fig. 5 for human and Additional file 2: Figures S25–S30 for other species). One should note that in the group of proteins not having an RBH, we also found a few highly expressed genes. This is due to the fact that some genes encode multiple structurally unrelated proteins which are assigned to different UniProt accession numbers. For example, the human GNAS complex locus (with Ensembl accession ID ENSG00000087460) is highly expressed in kidney (median FPKM > 330). In UniProtKB/Swiss-Prot this gene is represented by the accession numbers Q5JWF2, P84996, O95467 and P63092. For the first three accession numbers we found an RBH in the assembled kidney transcriptome while for P63092 we did not.

**Fig. 6:**
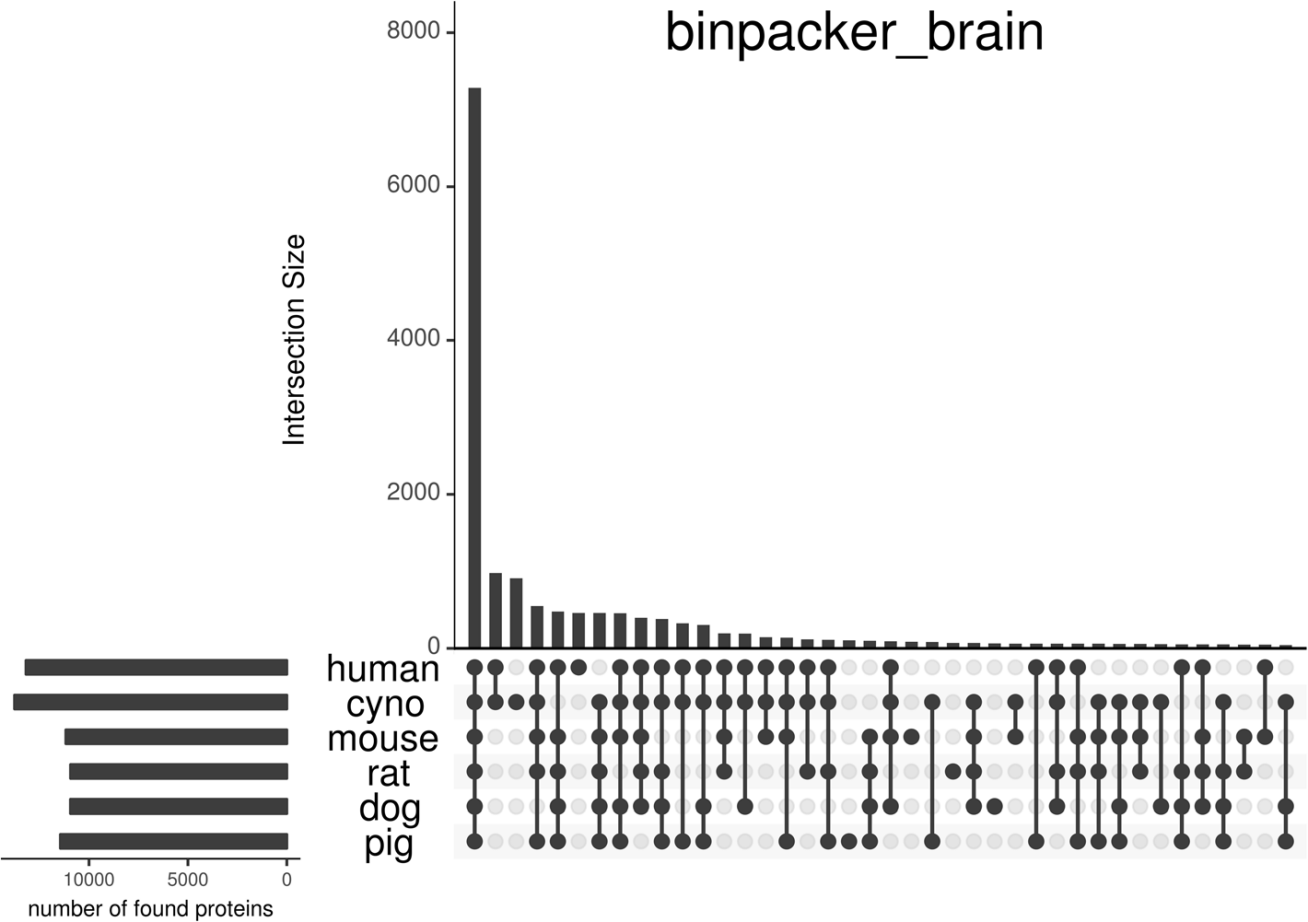
An UpSet [31] plot showing the number of proteins having a reciprocal best hit in each species and the intersections of these sets based on assemblies from brain data. Filled and connected dots indicate the intersection between the respective species’ sets

To get an idea of how appropriate it is to use human sequences as bait when searching non-human transcriptomes, we looked at the intersections between the RBHs in all investigated species and found that the majority of all proteins were consistently found as RBH in all six species (see Fig. 6). The second largest set consists of proteins having an RBH only in human and cynomolgus monkey*.* Interestingly, the third largest set are RBHs exclusively found in cynomolgus monkey*.*

In summary, the results of the reciprocal best hit BLAST analysis showed that the majority of known proteins are well-conserved across the investigated species and that the known human sequence can be used as a bait to robustly detect the corresponding orthologous cDNA sequence if the corresponding coding gene is expressed.

### Application of the generalised refinement pipeline

**Fig. 7:**
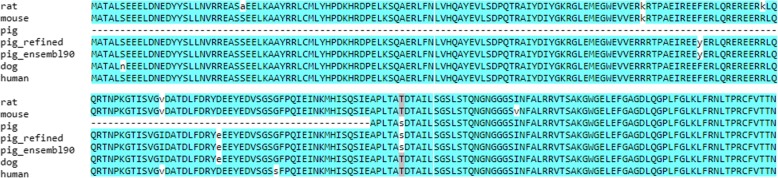
Multiple sequence alignment of the human protein DnaJ homolog subfamily C member 11 and its orthologues in rat, mouse, pig and dog (Ensembl 88). The refined sequence (pig_refined) and the pig sequence released with Ensembl 90 (pig_ensembl90) are also included in the alignment

To generalise the idea described in the previous section, we have implemented a fully automated, target-centric sequence refinement pipeline (see Fig. 2), called a&o-tool, in Nextflow [25]. As an example, we applied the a&o-tool to refine the pig sequence of the human protein DnaJ homolog subfamily C member 11 (DJC11_HUMAN, UniProt Accession Q9NVH1). The protein sequence is well conserved across human, mouse, rat and dog (> 94% protein sequence identity). However, the annotated orthologous pig sequence in Ensembl version 88 (ID ENSSSCP00000003669.2) lacked a stretch of 169 amino acids at the N-terminus of the protein. The a&o-tool was able to generate a full-length protein sequence which almost perfectly matches the human sequence and was confirmed by the updated sequence ENSSSCP00000003669.3 released with Ensembl version 90 (see Fig. 7).

**Fig. 8:**
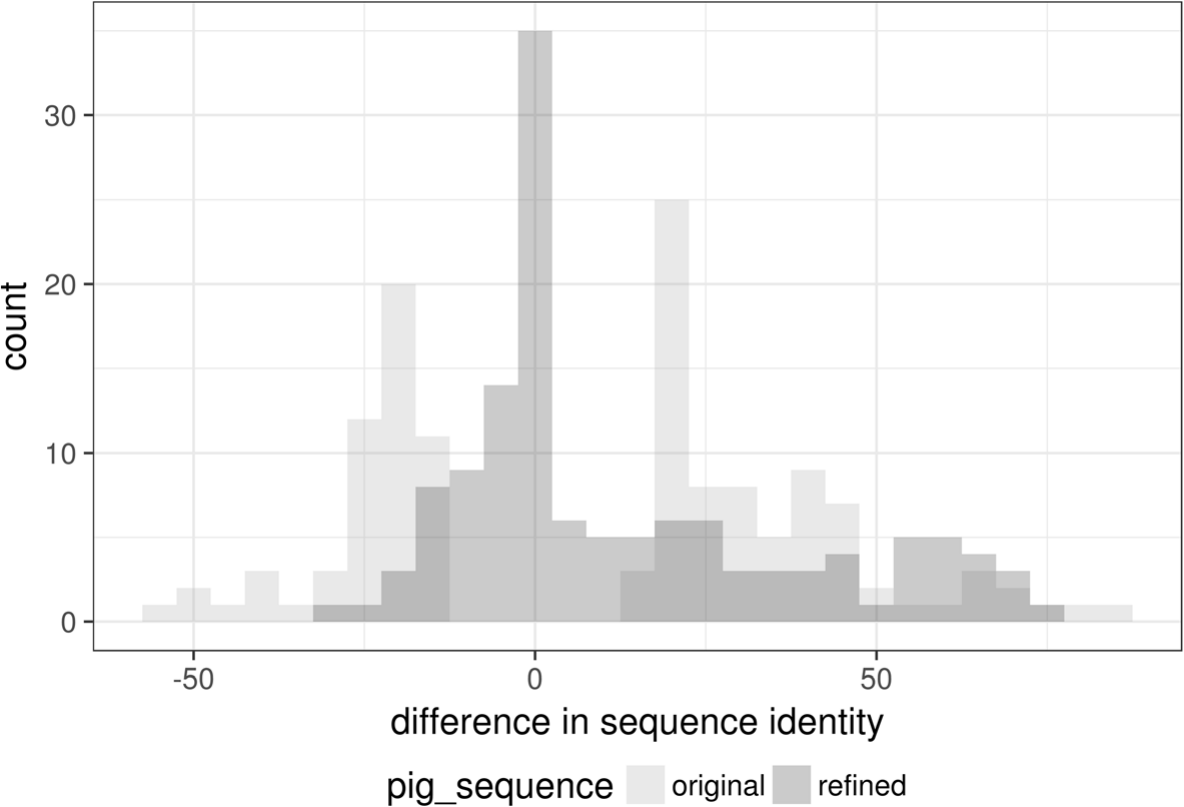
Distribution of the difference between target and query sequence identity before and after refinement for all pig proteins which were identified as presumably poorly annotated

To assess the performance of the a&o-tool on a larger scale we have also applied it to refine the sequence of the pig proteins which were identified as presumably poorly annotated (see Additional file 2: Figure S12) using human bait sequences. The a&o-tool generated results for 220 of the 293 proteins, the others either did not have an initial BLAST hit or no ORF was found in the respective contig. These 220 proteins were then filtered for those having a reciprocal best BLAST hit and for which neither the query identity nor the target identity varies by more than 3% between Ensembl annotation and the alignment with the Swiss-Prot sequence. The a&o-tool was able to achieve a mean decrease of 19.5% in absolute difference in sequence identity in 98 of these 131 (74.8%) filtered proteins (see Fig. 8).

## Discussion

The quality and completeness of protein sequences available in public databases are continuously improving, but one might still be faced with a target protein whose sequence is incomplete or not in accordance with orthologous, well conserved sequences from closely related species. With the a&o-tool we provide an easy-to-use tool to either validate or refine such sequences using RNA-Seq data. We have shown that the idea works well when comparing known human proteins to human transcriptome assemblies. However, sequence differences were observed which can be attributed to three reasons:

1. The majority of undetected sequences correspond to gene products of lowly expressed genes (see Fig. 5).
2. The known human protein sequences that we used for validation correspond to canonical (mostly full-length) isoforms. This simplifies the fact that many genes are expressed as different isoforms in different tissues leading to truncated or alternatively spliced gene products.
3. Although most known human sequences are well curated there are still a number of proteins affected by uncertainties and sequence conflicts or which may be missing. Approximately 7.4% of proteins have no prior evidence on the transcript level and/or are marked as inferred from homology, predicted or uncertain (data not shown, derived from UniProtKB annotation). Recently a team around Steven Salzberg released a “new, comprehensive catalogue of human genes and transcripts” [27] which contains roughly 1000 more protein-coding genes than UniProtKB/Swiss-Prot.

Due to the nature of short read RNA-Seq data one has to bear in mind that the contiguous sequences in the de novo assembled transcriptomes do not necessarily represent entire transcripts. On the one hand, this may lead to the identification of the correct, but too short contig which cannot be translated to a complete protein sequence, e.g. due to a missing initiation codon. On the other hand, the resulting predicted protein may be too long because of 5′ UTRs that contain initiation codons belonging to upstream open reading frames [28]. These issues can be tackled in two ways or a combination of them. Firstly, an additional filtering step, which removes short contigs or ones with low read coverage, could be incorporated after the assembly step. Secondly, long-read sequencing technologies such as PacBio SMRT sequencing or Oxford Nanopore could be used to replace or complement the short read RNA-Seq input with theoretically full length transcripts.

The results from validating our general idea via a reciprocal best hit BLAST search of known human proteins in tissue-specific transcriptome assemblies from several species show that the application of RNA-Seq data leads to a high and very consistent detection rate for a number of placental mammals which is linked to gene expression level as quantified by RNA-Seq (see Fig. 5 and Additional file 2: Figures S25–S30). It is, however, important to have high quality paired-end RNA-Seq data and reasonable mRNA expression of the target gene. Proteins which are found as RBHs are generally well covered by an HSP with high sequence identity. The observed rank order of tissue specific hit rates is in line with the percentage of expressed proteins as reported on the Human Protein Atlas website, i.e. 74% of all considered human proteins are expressed in brain tissue, 68% in kidney and 59% in liver [29].

The importance of high quality RNA-Seq data is also emphasised by the fact that the hit rate achieved in the search of cynomolgus monkey assemblies using human bait sequences is even greater than that of human vs. human. Furthermore, the HSPs obtained in the reciprocal BLAST search of cynomolgus monkey assemblies and known human proteins show a higher sequence identity and coverage than those resulting from comparisons of the other non-human species to the known human proteins. This might be due to greater RNA quality and sequencing depth of the in-house cynomolgus monkey study. For cynomolgus monkey the median RNA integrity number (RIN) across all samples was 8.7 and the median number of uniquely mapped reads is 4.96e+ 07 while in the Fushan data for mouse, rat, pig and dog the median number of uniquely mapped reads is 1.36e+ 07 (median of per species medians) and the median RIN value is 7.5, thus significantly lower. With 1.15e+ 07 uniquely mapped reads (again median across samples) for the human data from the Human Protein Atlas there are even less reads than in the other data sets.

The high number of RBHs found in all three tissues highlights the potential to detect tissue-specific proteins. Thus, by applying the a&o-tool to assemblies from different tissues, one can find functionally important proteins in tissues of interest.

When investigating target proteins which are part of families of highly similar proteins, e.g. kinases, one has to pay particular attention in interpreting the results of our approach. The same contig might be found as best match for several members of the family and will thus lead to the same translated protein sequence for multiple, similar bait proteins.

Finally, we applied the a&o-tool to all genes which we identified as presumably poorly annotated in pig. Overall, we found that the median difference in sequence identity was shifted to 0 and using the known sequence from human as bait lead to improved sequence information in the majority of cases. Therefore, we hope that this target-centric pipeline is a valuable tool for target discovery and validation.

## Conclusions

In summary, we were able to show that exploiting RNA-Seq data and sequence information from closely related species leads to improved protein sequences for species with poorly or no annotated sequence for a specific target protein. With the a&o-tool we provide an automated pipeline to perform this refinement task. The major prerequisite for using the a&o-tool is high quality sequencing data.

## Availability and requirements

The Nextflow pipeline is available.

**Project name:** a&o-tool

**Project home page:** https://github.com/Julia-F-S/a-o-tool

**Archived version:** 10.5281/zenodo.1451221

**Operating system(s):** Linux

**Programming language:** Nextflow

**Other requirements:** Docker

**License:** GNU GPL 3.0

**Any restrictions to use by non-academics:** None

## Additional files

Additional file 1: An overview of the RNA-Seq samples. (XLSX 12 kb)

Additional file 2: Additional figures. (PDF 2.57 mb)

Additional file 3: TransRate results for all investigated species. (CSV 2 kb)

## Abbreviations

BLAST: Basic local alignment search tool
CHO: Chinese hamster ovary cells
GEO: Gene Expression Omnibus
HSP: High-scoring segment pair
MSA: Multiple sequence alignment
ORF: Open reading frame
RBH: Reciprocal best hit BLAST
SMRT: Single-molecule real time
