# Supplementary material for: Exploiting orthology and de novo transcriptome assembly to refine target sequence information
Source: BMC Med Genomics. 2019 May 23;12:69. doi: 10.1186/s12920-019-0524-5 (PMC6533699; doi:10.1186/s12920-019-0524-5)
Supplement: Supplementary file 2 — Additional figures. (PDF 2.57 mb) [file 12920_2019_524_MOESM2_ESM.pdf]

Supplementary Figures

Methods - RNA-Seq data mapping, counting and normalization

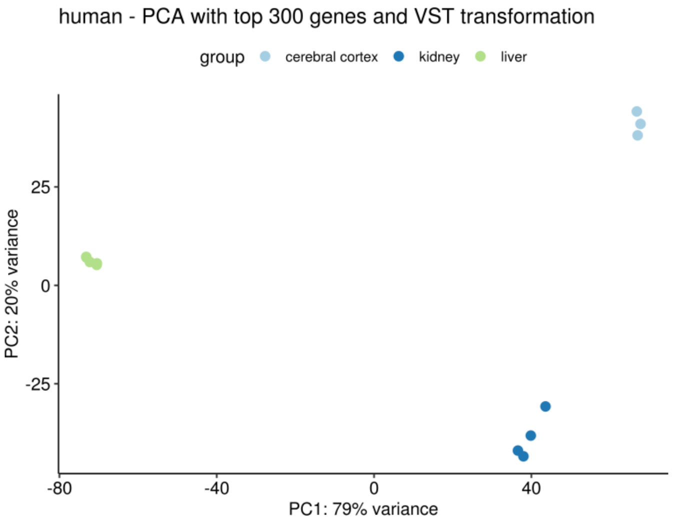

Figure S1 Principal component analysis of variance-stabalisation transformed (VST) read counts of the top 300 genes in human tissue samples.

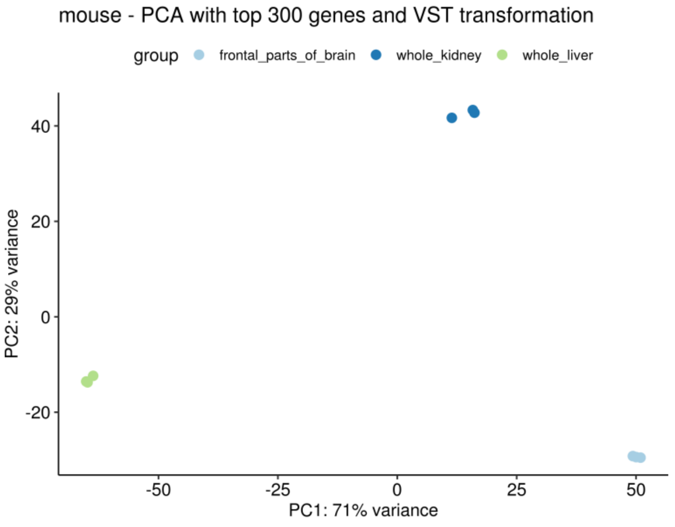

Figure S2 Principal component analysis of variance-stabalisation transformed (VST) read counts of the top 300 genes in mouse tissue samples.

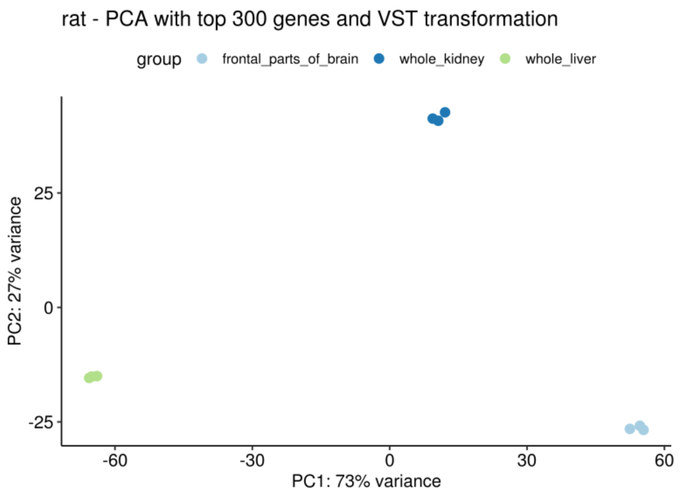

Figure S3 Principal component analysis of variance-stabalisation transformed (VST) read counts of the top 300 genes in rat tissue samples.

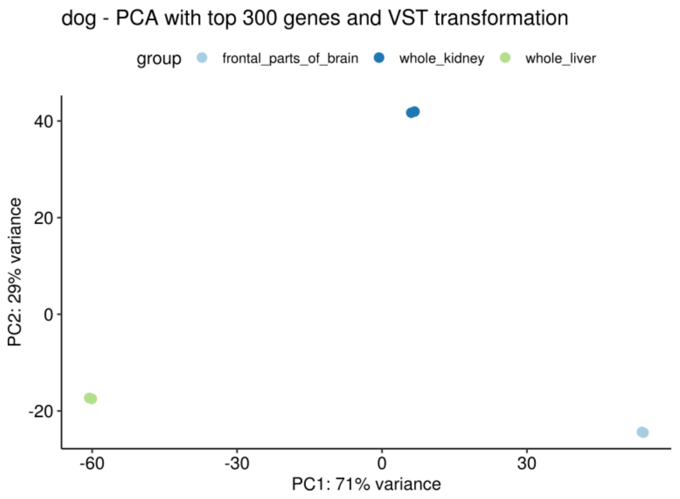

Figure S4 Principal component analysis of variance-stabalisation transformed (VST) read counts of the top 300 genes in dog tissue samples.

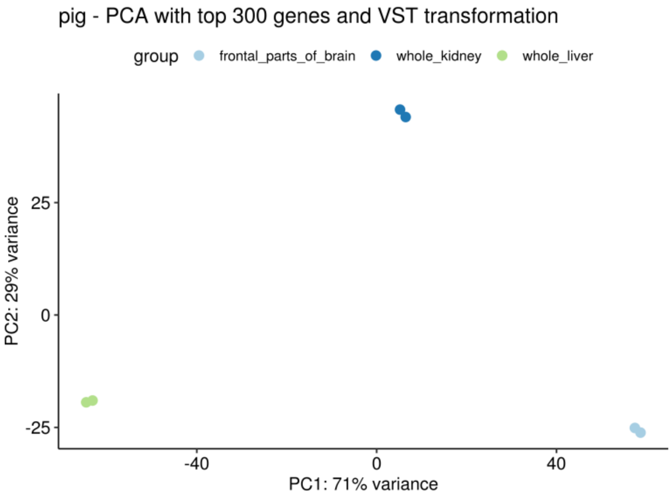

Figure S5 Principal component analysis of variance-stabalisation transformed (VST) read counts of the top 300 genes in pig tissue samples.

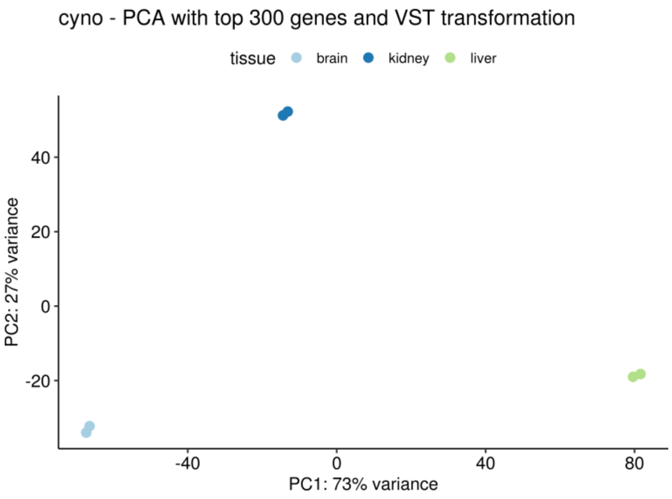

Figure S6 Principal component analysis of variance-stabalisation transformed (VST) read counts of the top 300 genes in cynomolgus monkey tissue samples.

Methods – number of genes with potential need for improvement

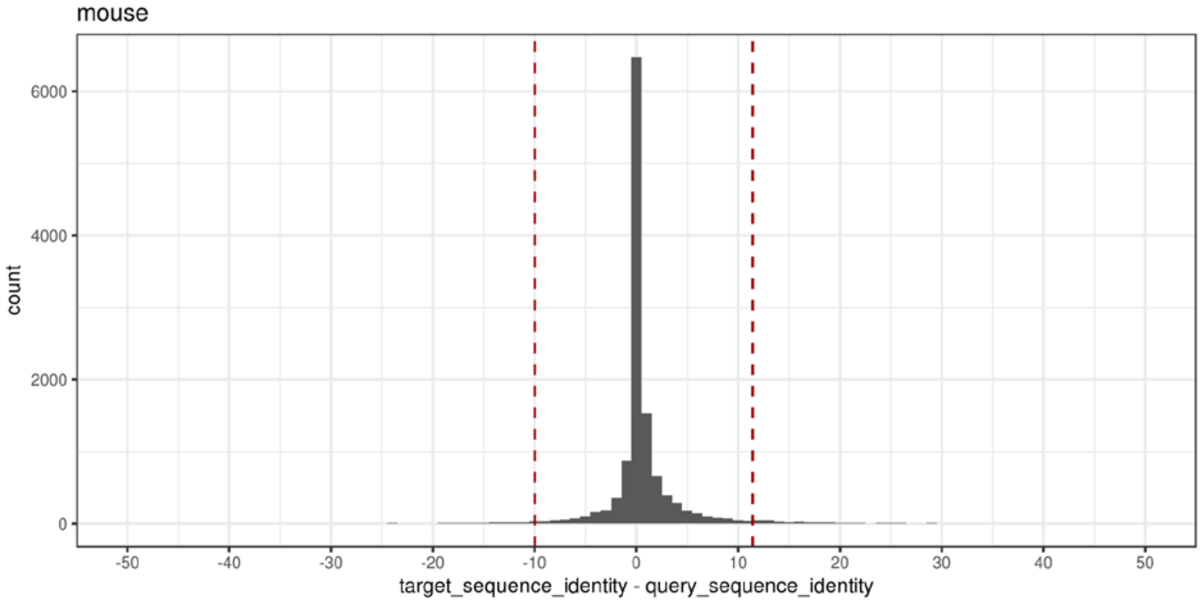

Figure S7 Distribution of the difference in sequence identites for all human genes having a one-to-one orthologue in mouse. The target sequence identity corresponds to the the percentage of orthologous sequence matching the human sequence in the amino acid sequence alignment and query identity is the percentage of human sequence matching the orthologous sequence. Dashed red lines mark the threshold (mean +/- 2 times standard deviation) for considering a gene for refinement.

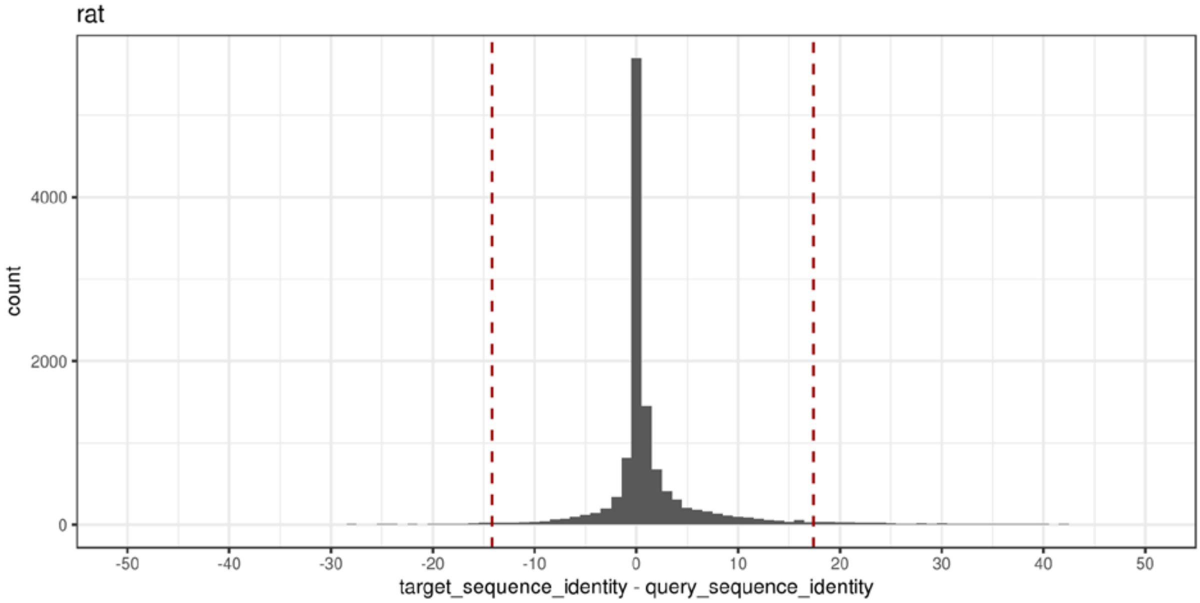

Figure S8 Distribution of the difference in sequence identites for all human genes having a one-to-one orthologue in rat. The target sequence identity corresponds to the the percentage of orthologous sequence matching the human sequence in the amino acid sequence alignment and query identity is the percentage of human sequence matching the orthologous sequence. Dashed red lines mark the threshold (mean +/- 2 times standard deviation) for considering a gene for refinement.

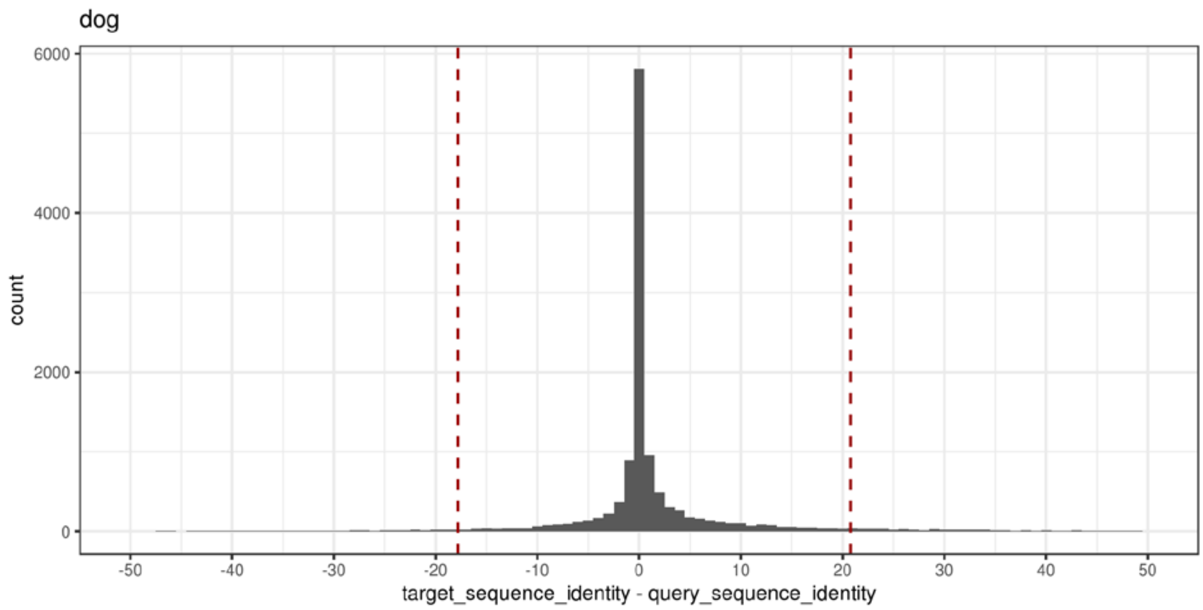

Figure S9 Distribution of the difference in sequence identites for all human genes having a one-to-one orthologue in dog. The target sequence identity corresponds to the the percentage of orthologous sequence matching the human sequence in the amino acid sequence alignment and query identity is the percentage of human sequence matching the orthologous sequence. Dashed red lines mark the threshold (mean +/- 2 times standard deviation) for considering a gene for refinement.

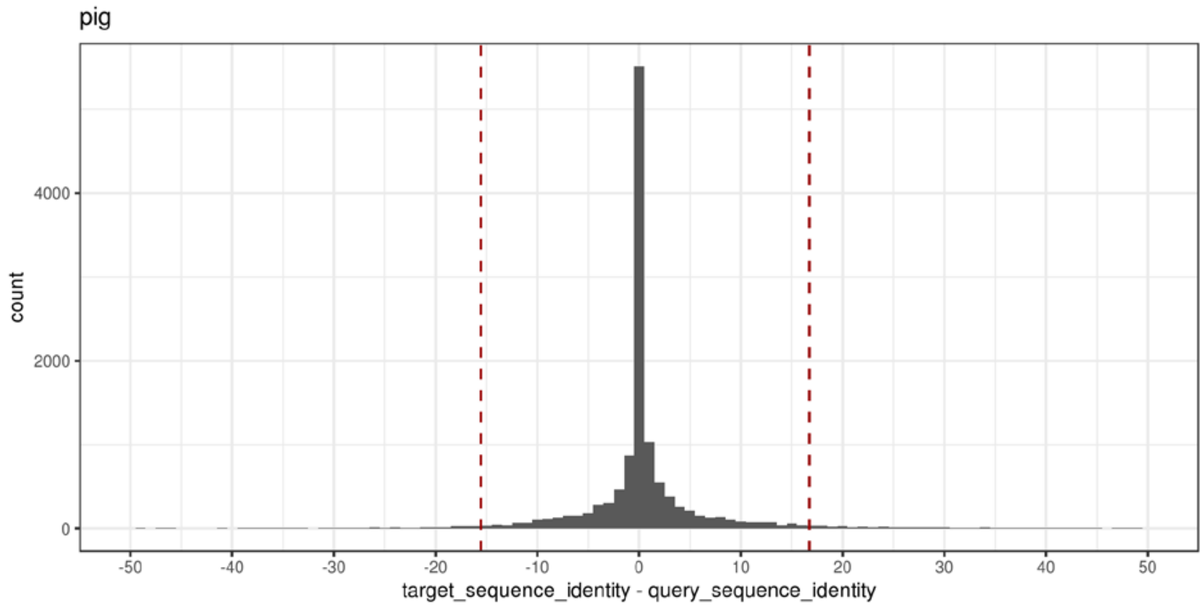

Figure S10 Distribution of the difference in sequence identites for all human genes having a one-to-one orthologue in pig. The target sequence identity corresponds to the the percentage of orthologous sequence matching the human sequence in the amino acid sequence alignment and query identity is the percentage of human sequence matching the orthologous sequence. Dashed red lines mark the threshold (mean +/- 2 times standard deviation) for considering a gene for refinement.

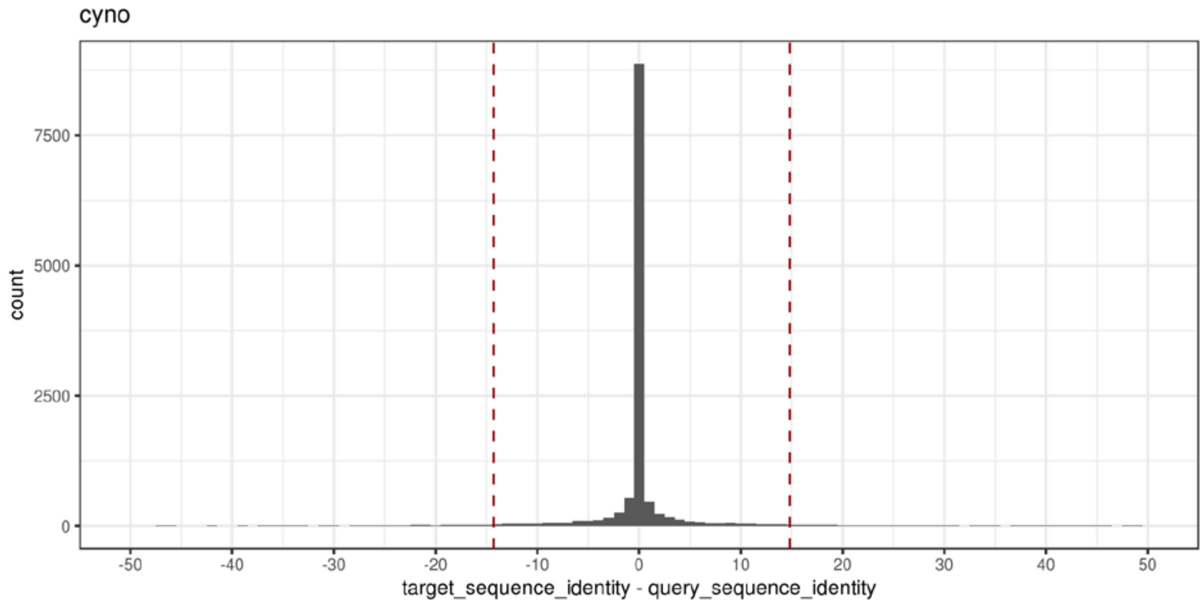

Figure S11 Distribution of the difference in sequence identites for all human genes having a one-to-one orthologue in cynomolgus monkey. The target sequence identity corresponds to the the percentage of orthologous sequence matching the human sequence in the amino acid sequence alignment and query identity is the percentage of human sequence matching the orthologous sequence. Dashed red lines mark the threshold (mean +/- 2 times standard deviation) for considering a gene for refinement.

Results – number of genes with potential need for improvement

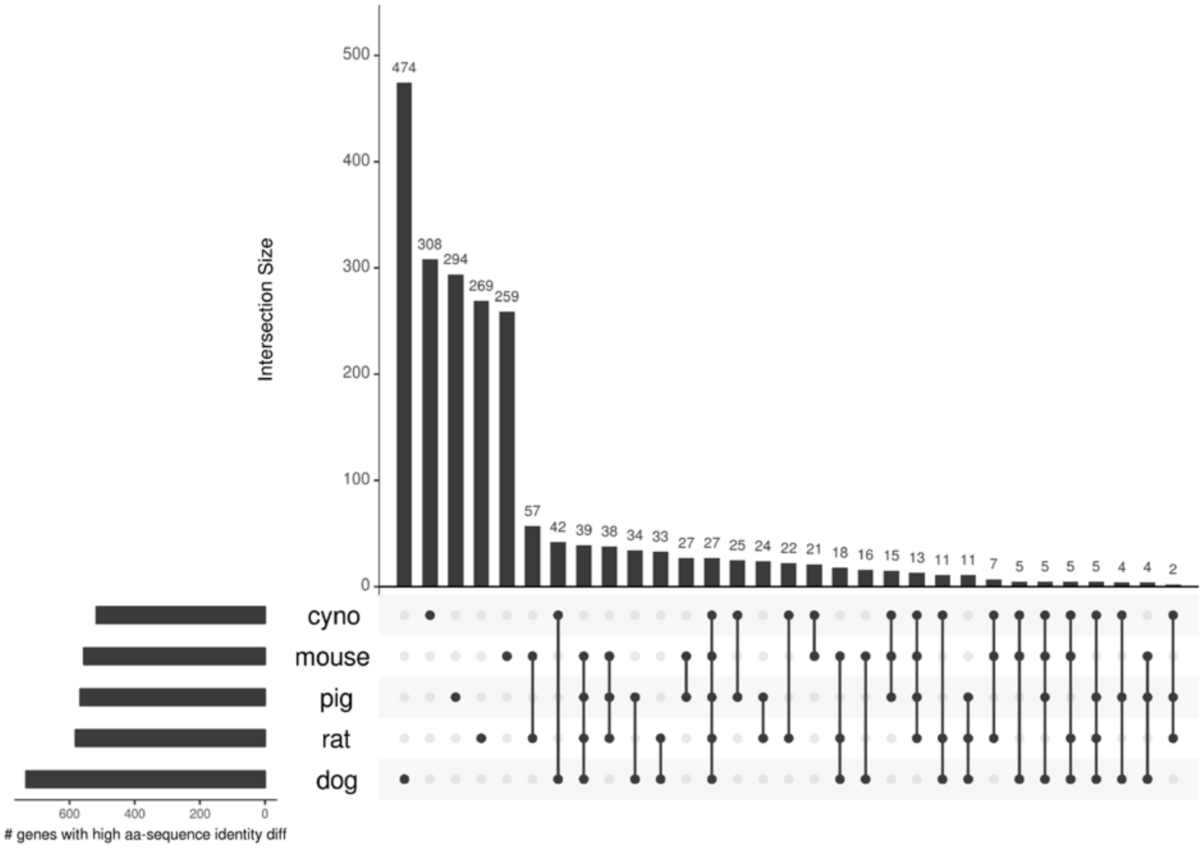

Figure S12 The set of genes with a high difference in sequence identity in Ensembl 92 was determined for each species and intersections are visualised in an UpSet plot. A non-human gene/protein was considered to be improvable if its absolute difference in sequence identity is greater than the mean difference in sequence identities over all genes in the respective species plus two times the corresponding standard deviation.

Results – Validation

Cumulative Coverage plots

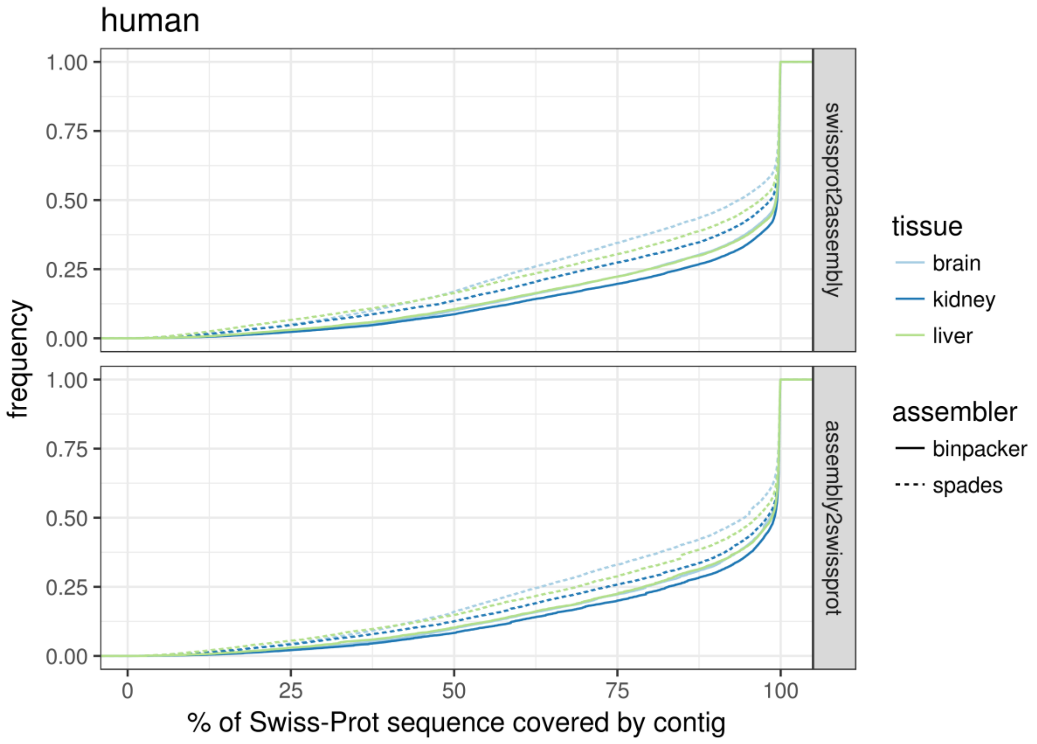

Figure S13 Cumulative distribution of the coverage of the known human protein.

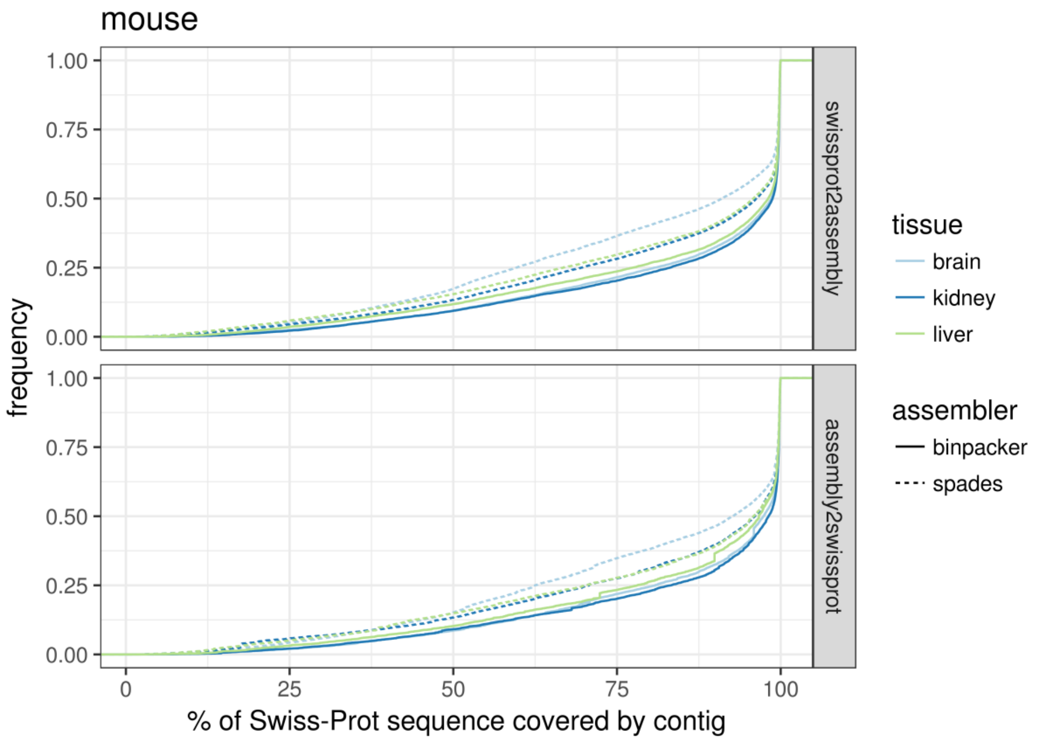

Figure S14 Cumulative distribution of the coverage of the known human protein.

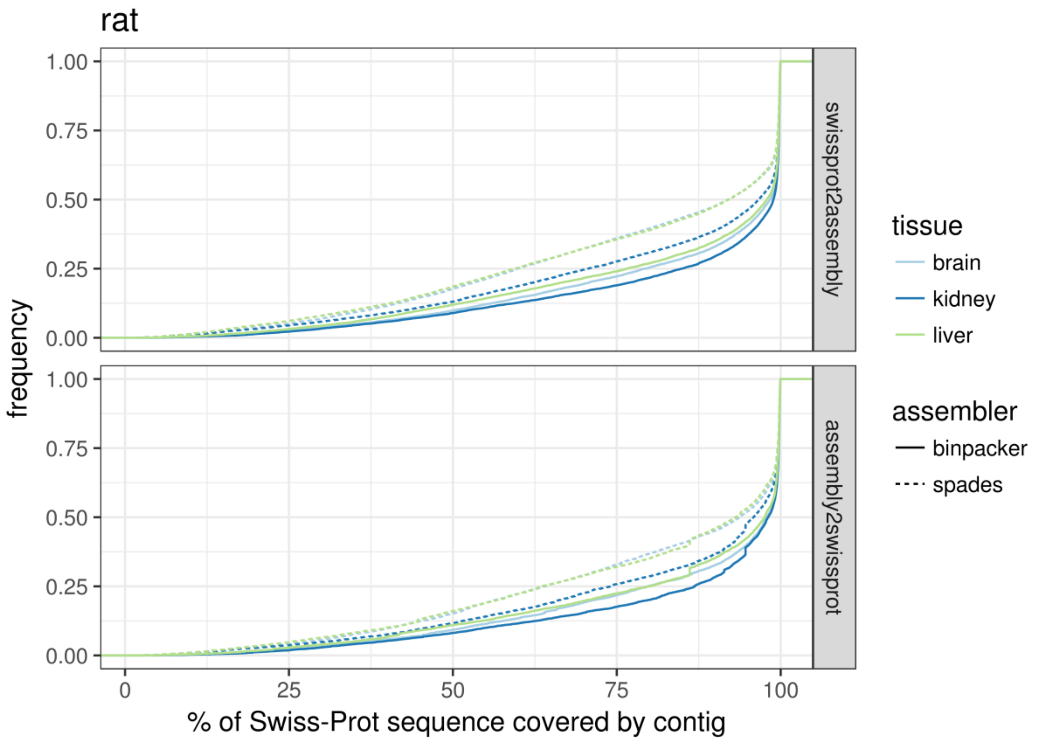

Figure S15 Cumulative distribution of the coverage of the known human protein.

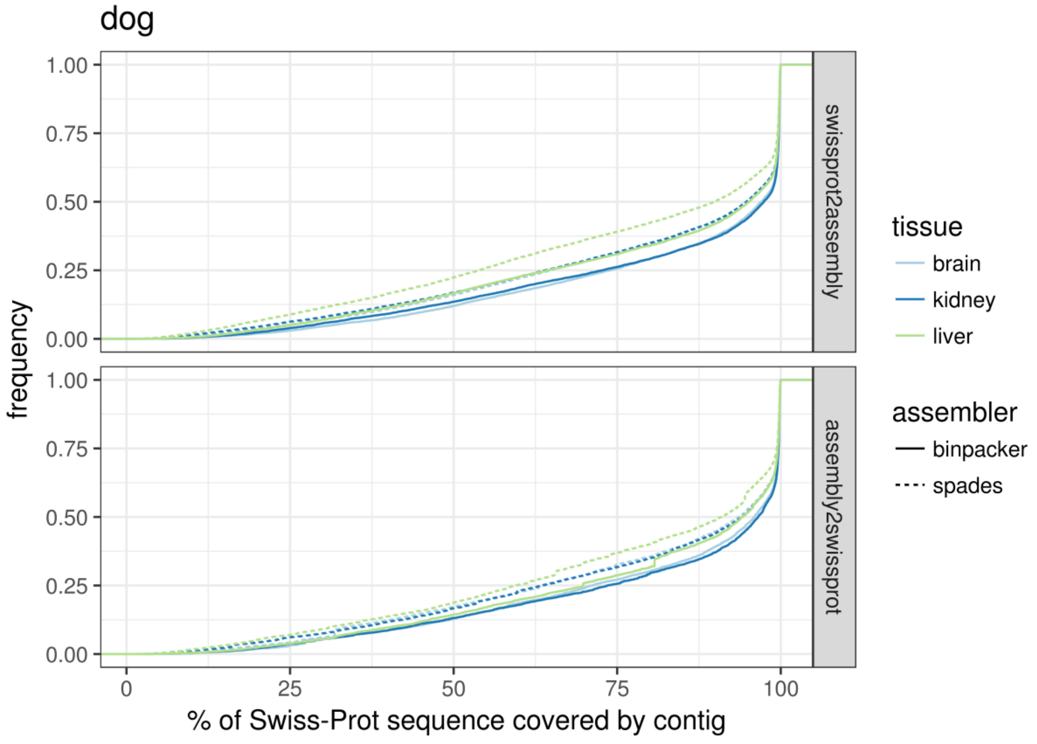

Figure S16 Cumulative distribution of the coverage of the known human protein.

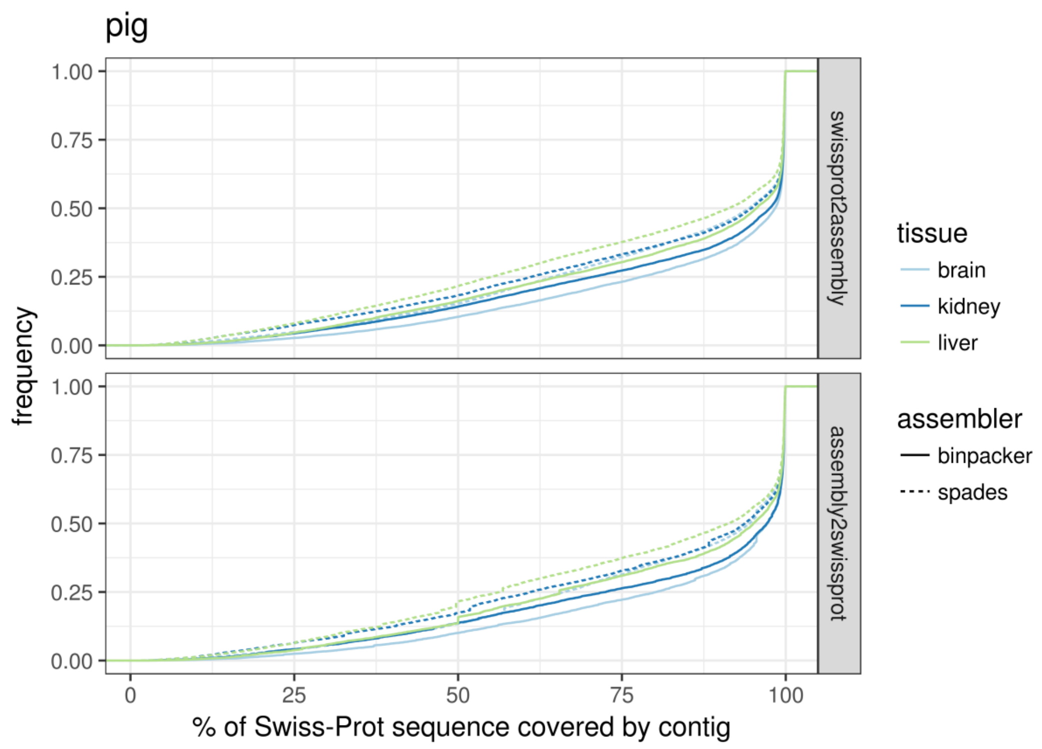

Figure S17 Cumulative distribution of the coverage of the known human protein.

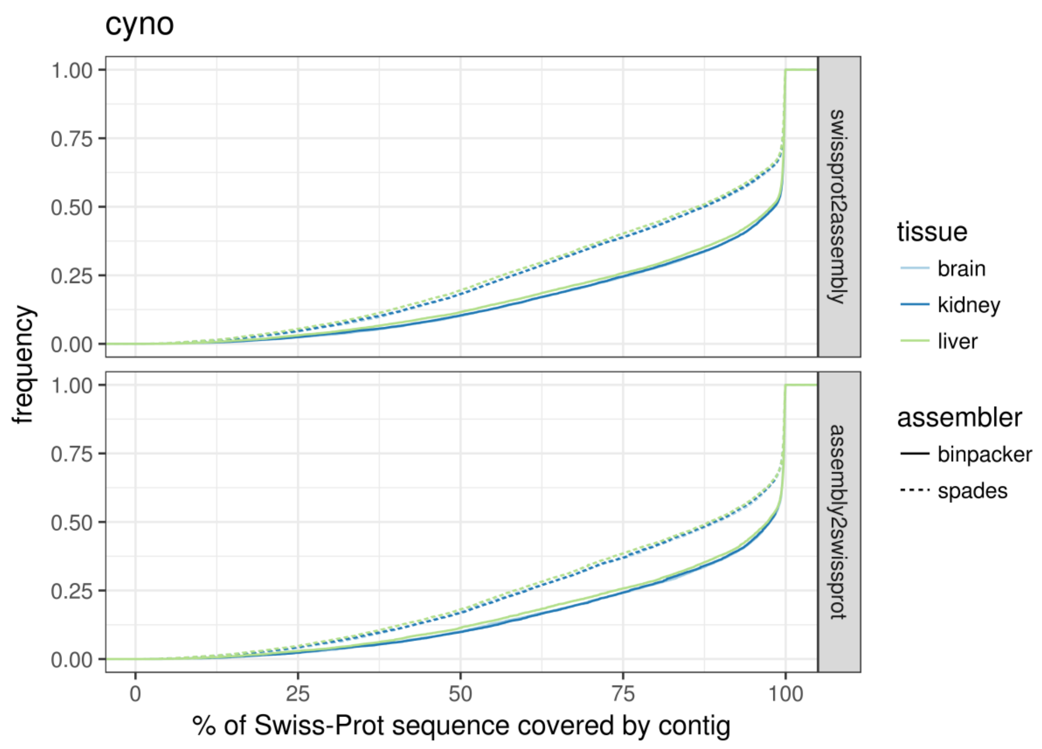

Figure S18 Cumulative distribution of the coverage of the known human protein.

Cumulative Identity plots

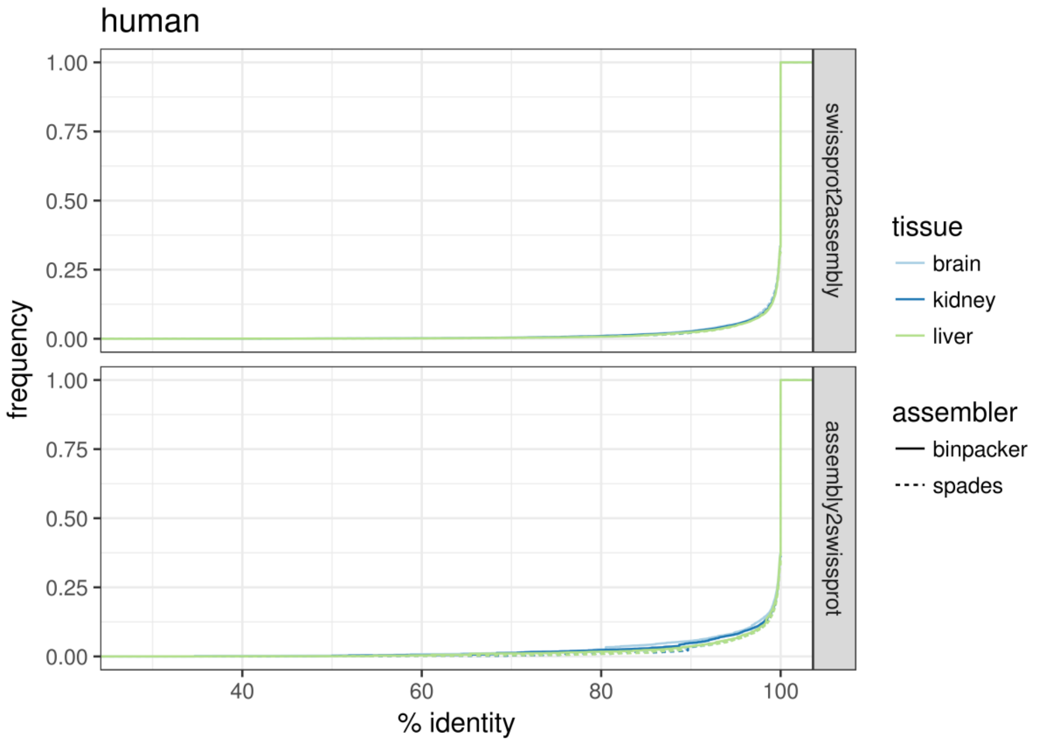

Figure S19 Cumulative density of the percent identity reported by BLAST.

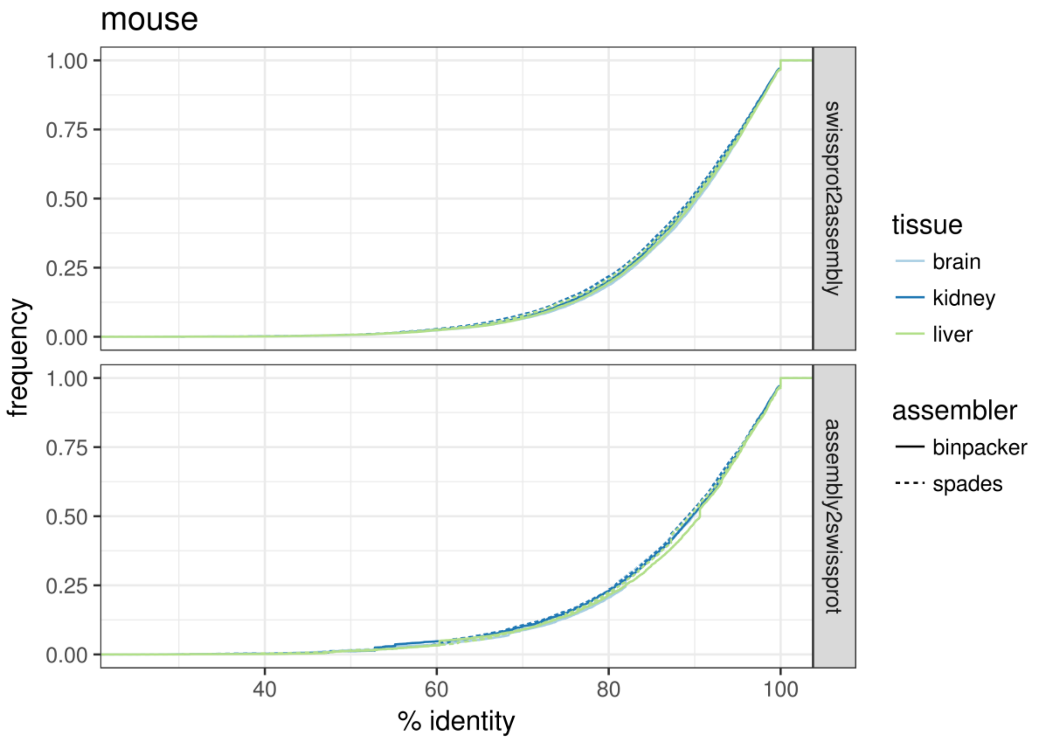

Figure S20 Cumulative density of the percent identity reported by BLAST.

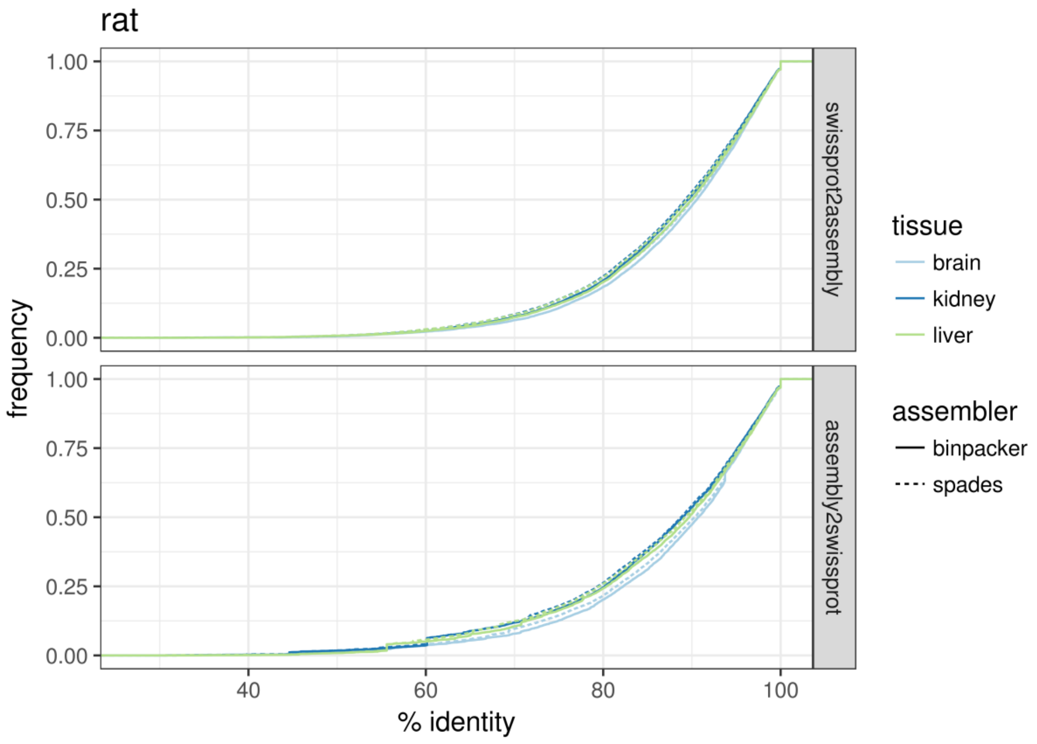

Figure S21 Cumulative density of the percent identity reported by BLAST.

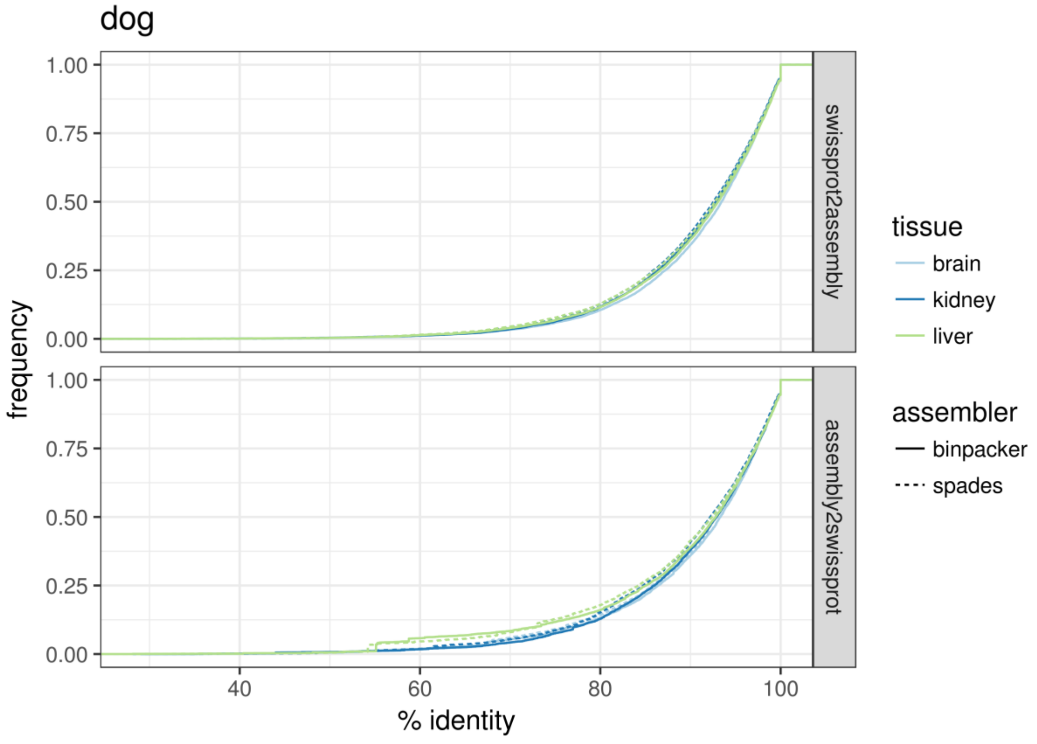

Figure S22 Cumulative density of the percent identity reported by BLAST.

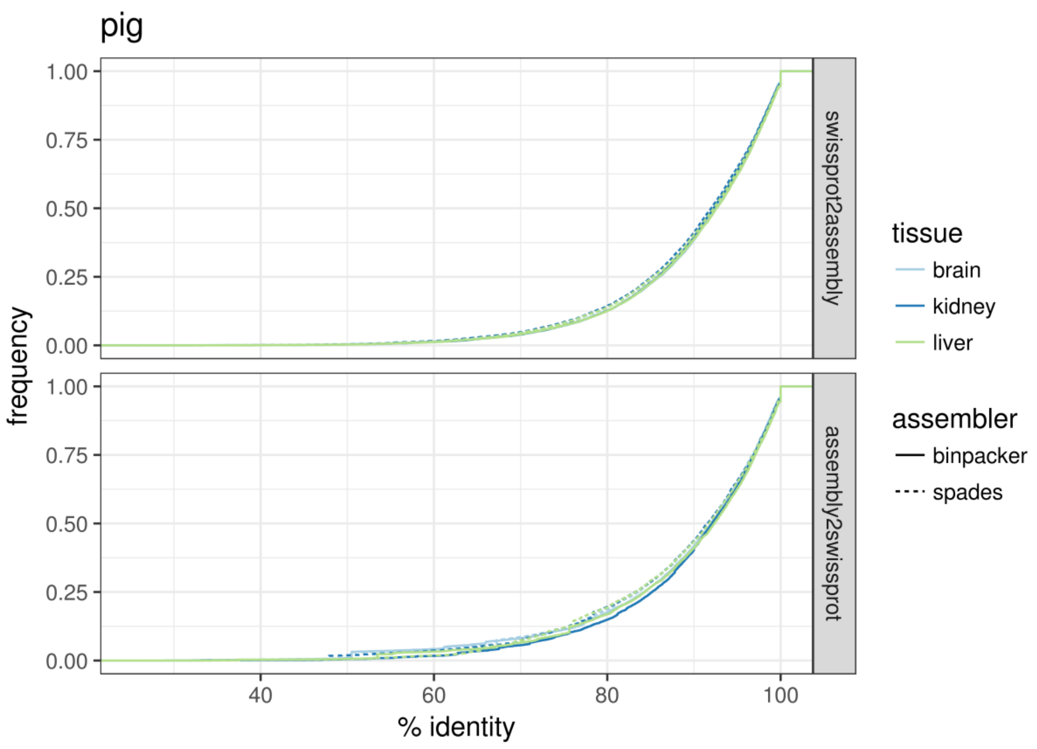

Figure S23 Cumulative density of the percent identity reported by BLAST.

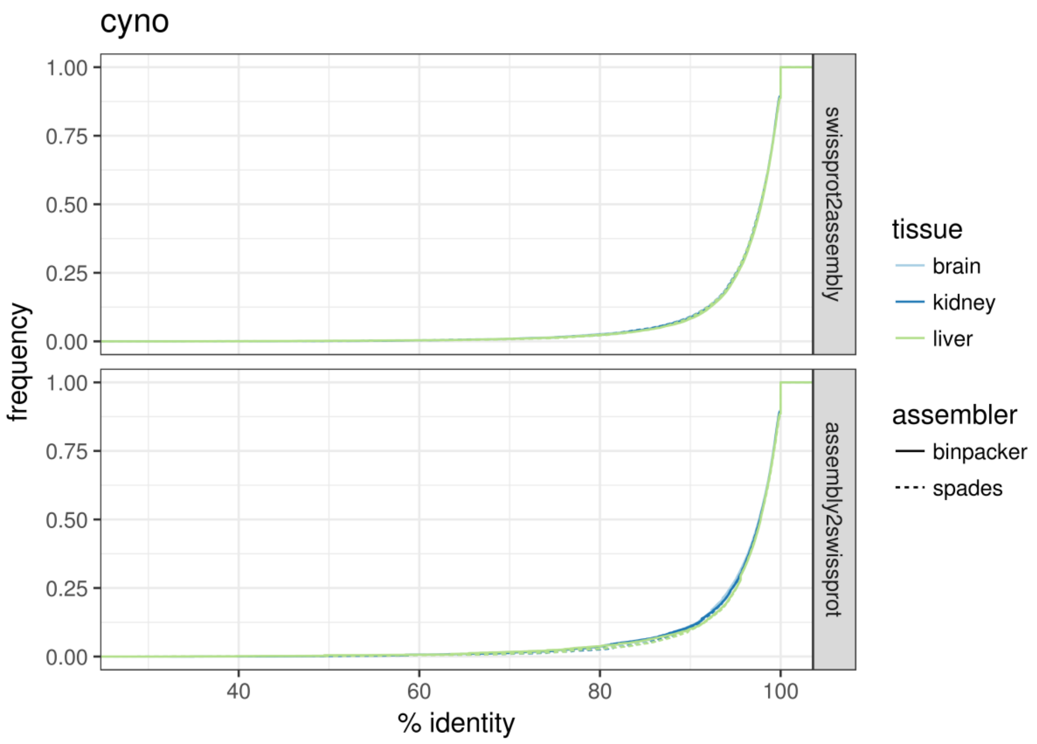

Figure S24 Cumulative density of the percent identity reported by BLAST.

Expression plots

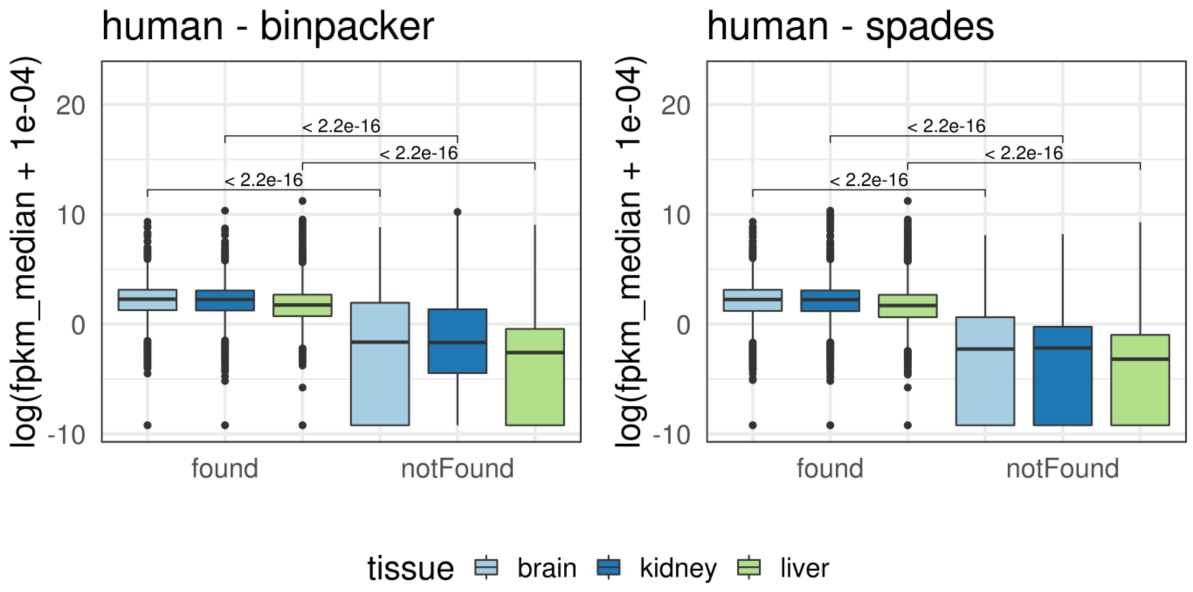

Figure S25 Expression levels in human between proteins with a reciprocal best BLAST hit in BinPacker (left) and SPAdes (right) assemblies (found) and those without (notFound). Tissue-specific pairwise significance has been determined with a Wilcoxon rank sum test.

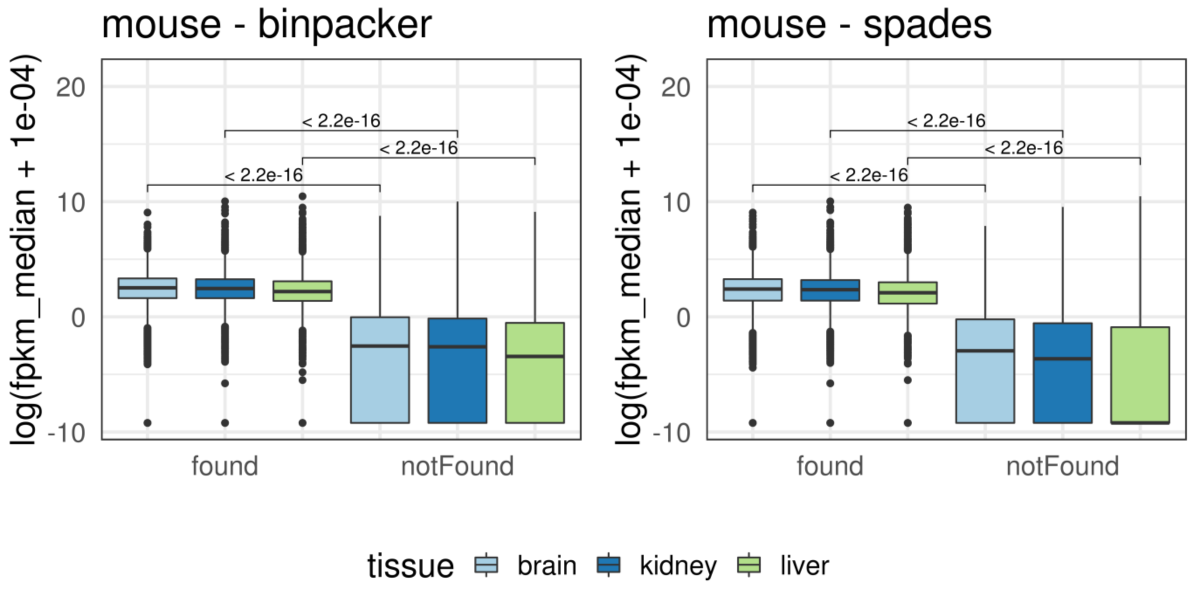

Figure S26 Expression levels in mouse between proteins with a reciprocal best BLAST hit in BinPacker (left) and SPAdes (right) assemblies (found) and those without (notFound). Tissue-specific pairwise significance has been determined with a Wilcoxon rank sum test.

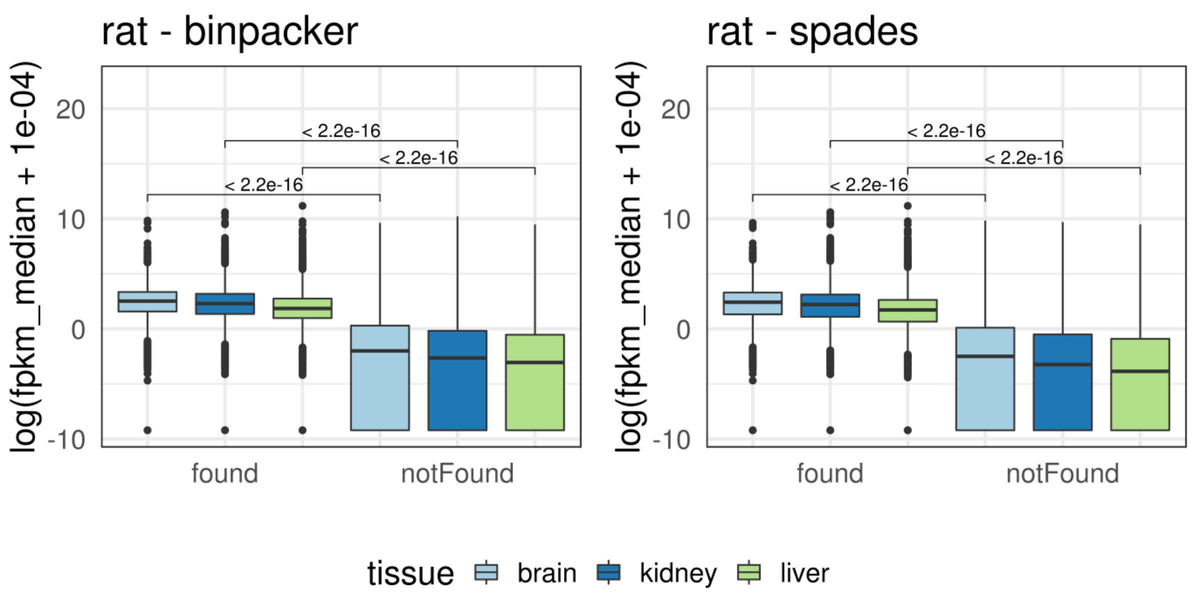

Figure S27 Expression levels in rat between proteins with a reciprocal best BLAST hit in BinPacker (left) and SPAdes (right) assemblies (found) and those without (notFound). Tissue-specific pairwise significance has been determined with a Wilcoxon rank sum test.

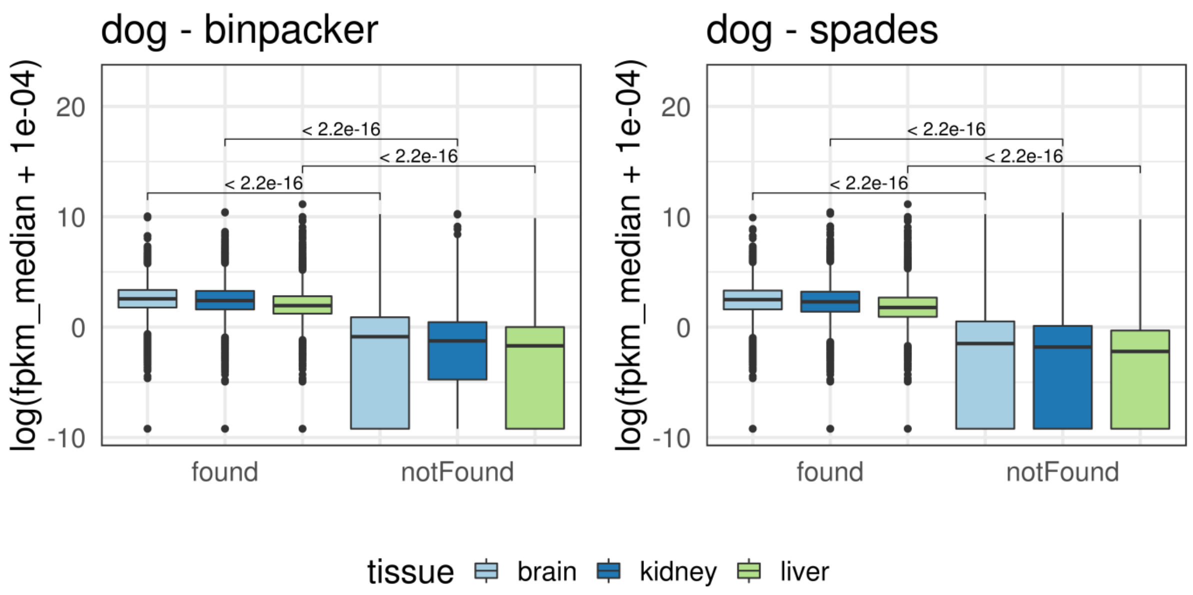

Figure S28 Expression levels in dog between proteins with a reciprocal best BLAST hit in BinPacker (left) and SPAdes (right) assemblies (found) and those without (notFound). Tissue-specific pairwise significance has been determined with a Wilcoxon rank sum test.

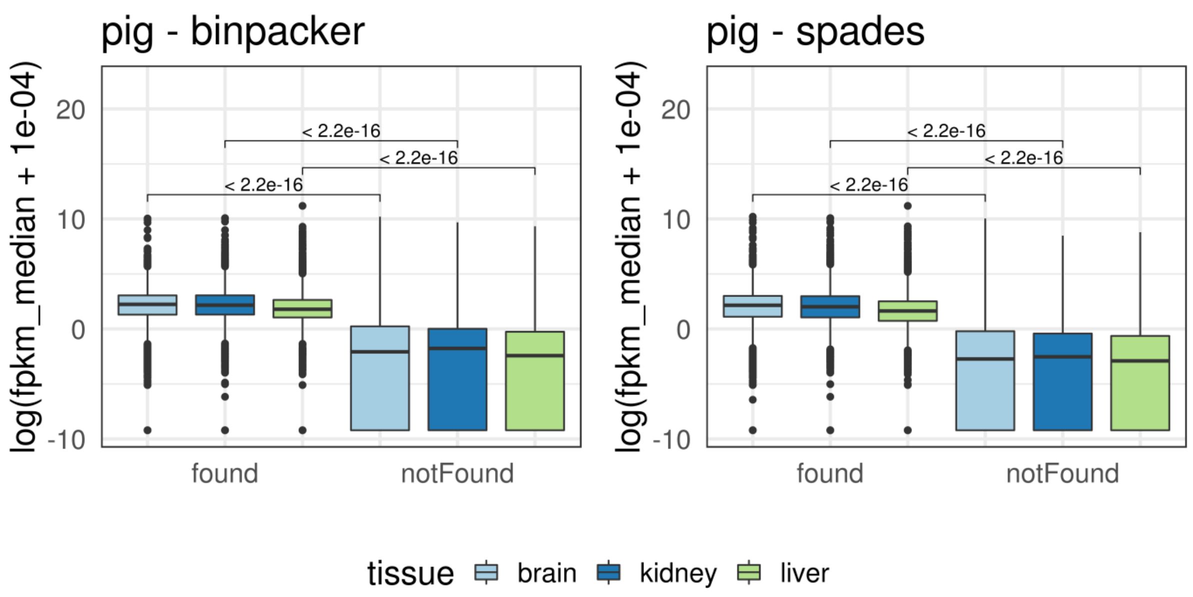

Figure S29 Expression levels in pig between proteins with a reciprocal best BLAST hit in BinPacker (left) and SPAdes (right) assemblies (found) and those without (notFound). Tissue-specific pairwise significance has been determined with a Wilcoxon rank sum test.

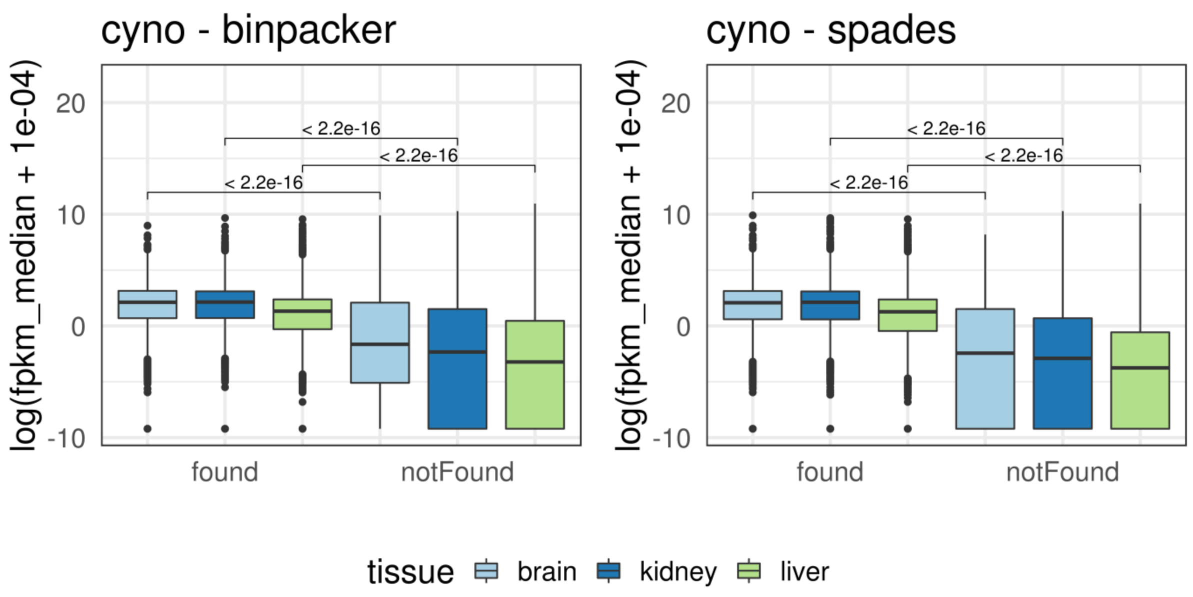

Figure S30 Expression levels in cynomolgus monkey between proteins with a reciprocal best BLAST hit in BinPacker (left) and SPAdes (right) assemblies (found) and those without (notFound). Tissue-specific pairwise significance has been determined with a Wilcoxon rank sum test.
